# Single-cell RNA sequencing reveals intrinsic and extrinsic regulatory heterogeneity in yeast responding to stress

**DOI:** 10.1371/journal.pbio.2004050

**Published:** 2017-12-14

**Authors:** Audrey P. Gasch, Feiqiao Brian Yu, James Hose, Leah E. Escalante, Mike Place, Rhonda Bacher, Jad Kanbar, Doina Ciobanu, Laura Sandor, Igor V. Grigoriev, Christina Kendziorski, Stephen R. Quake, Megan N. McClean

**Affiliations:** 1 Laboratory of Genetics, University of Wisconsin–Madison, Madison, Wisconsin, United States of America; 2 Great Lakes Bioenergy Research Center, University of Wisconsin–Madison, Madison, Wisconsin, United States of America; 3 Department of Bioengineering, Stanford University, Stanford, California, United States of America; 4 Department of Biostatistics and Medical Informatics, University of Wisconsin–Madison, Madison, Wisconsin, United States of America; 5 Department of Energy Joint Genome Institute, Walnut Creek, California, United States of America; 6 Chan Zuckerberg Biohub, San Francisco, California, United States of America; 7 Department of Biomedical Engineering, University of Wisconsin–Madison, Madison, Wisconsin, United States of America; The Hebrew University of Jerusalem, Israel

## Abstract

From bacteria to humans, individual cells within isogenic populations can show significant variation in stress tolerance, but the nature of this heterogeneity is not clear. To investigate this, we used single-cell RNA sequencing to quantify transcript heterogeneity in single *Saccharomyces cerevisiae* cells treated with and without salt stress to explore population variation and identify cellular covariates that influence the stress-responsive transcriptome. Leveraging the extensive knowledge of yeast transcriptional regulation, we uncovered significant regulatory variation in individual yeast cells, both before and after stress. We also discovered that a subset of cells appears to decouple expression of ribosomal protein genes from the environmental stress response in a manner partly correlated with the cell cycle but unrelated to the yeast ultradian metabolic cycle. Live-cell imaging of cells expressing pairs of fluorescent regulators, including the transcription factor Msn2 with Dot6, Sfp1, or MAP kinase Hog1, revealed both coordinated and decoupled nucleocytoplasmic shuttling. Together with transcriptomic analysis, our results suggest that cells maintain a cellular filter against decoupled bursts of transcription factor activation but mount a stress response upon coordinated regulation, even in a subset of unstressed cells.

## Author summary

Genetically identical cells growing in the same environment can vary in their cellular state and behavior. Such heterogeneity may explain why some cells in an isogenic population can survive sudden severe environmental stress whereas other cells succumb. Cell-to-cell variation in gene expression has been linked to variable stress survival, but how and why transcript levels vary across the transcriptome in single cells is only beginning to emerge. Here, we used single-cell RNA sequencing (scRNA-seq) to measure cell-to-cell heterogeneity in the transcriptome of budding yeast (*Saccharomyces cerevisiae*). We find surprising patterns of variation across known sets of transcription factor targets, indicating that cells vary in their transcriptome profile both before and after stress exposure. scRNA-seq analysis combined with live-cell imaging of transcription factor activation dynamics revealed some cells in which the stress response was coordinately activated and other cells in which the traditional response was decoupled, suggesting unrecognized regulatory nuances that expand our understanding of stress response and survival.

## Introduction

When adversity strikes, it is often the case that some cells in an isogenic population survive whereas others do not. Such phenotypic heterogeneity has been observed in isogenic microbes exposed to environmental stress as well as normal and malignant human cells surviving chemotherapy drugs [1–7]. While genetic mutations can produce cells with heritably high stress tolerance, in many cases, the heterogeneity is transiently induced by epigenetic processes [8,9]. For example, some isogenic cells within cultures of *Saccharomyces cerevisiae* can survive extreme heat stress, whereas most cells in the culture cannot [10]. Stress-tolerant individuals may be in an altered state, because they often display transiently reduced growth and markers of the stress response [1,10–12]. But whether this state mimics that of stress-treated cells that have fully mounted a stress response or instead emerges from partial responses or stochastic events is unclear. Understanding what gives rise to cell-to-cell heterogeneity in stress survival has broad applications, from treating pathogenic microbial infections to blocking drug-resistant human metastases.

In several systems, variation in stress tolerance can be traced to heterogeneous expression of defense genes. Graded expression of the stress-responsive *TSL1* gene in unstressed yeast quantitatively predicts how well individual cells in a culture will survive severe heat [10]. In some cases, such variation is “intrinsic” to the gene promoter: many defense genes are transcribed through TATA-dependent promoters [13,14], which produce stochastic transcriptional bursts proposed to play a role in bet-hedging [15–19]. Stochastic fluctuations can also be intrinsic to specific regulators, such that their target genes vary coherently but without a widespread cellular response [20]. But “extrinsic” variation in the cellular system, e.g., activation of the broader upstream signaling response or transition through other physiological states, likely plays an important role. Several studies have interrogated tagged protein abundance in yeast to explore noise in single or paired protein abundances [15,19,21–23]. Stewart-Ornstein et al. showed that targets of the yeast “general-stress” responsive transcription factor Msn2 often behave coordinately in single cells, suggesting concerted activation of the entire regulon even in the absence of added stress [22,23]. Targets of several other transcription factors also showed coordinate behavior across single cells, suggesting that the variation may emerge from stochastic activation of Protein Kinase A (PKA), a common upstream regulator of those factors [22,23].

In response to acute stress, Msn2 is activated as part of a broader signaling network that regulates the Environmental Stress Response (ESR), a common transcriptomic response triggered by diverse stresses [24,25]. The ESR includes induced expression of approximately 300 defense genes, regulated in part by Msn2 and its paralog Msn4, which is coordinated with repression of approximately 600 genes encoding ribosomal proteins (RPs) and factors involved in ribosome biogenesis (RiBi) and other processes. RP and RiBi genes are thought to be highly transcribed in actively growing cells, but repressed in response to stress through release of the RP activator Sfp1 or recruitment of the RiBi transcriptional repressors Dot6/Tod6 and histone deacetylases [26–30]. Activation of the ESR after mild stress can impart increased tolerance to subsequent stress, known as acquired stress resistance [31–33]. In some cases, the ESR program also correlates with reduced growth rate, most notably in nutrient-restricted chemostats and in slow-growing mutants potentially experiencing internal stress [34–38]. In fact, O’Duibhir proposed that the ESR may simply be a byproduct of cell-cycle phase, since slow-growing mutants with prolonged G1 phase display an ESR-like transcriptome profile [38]. In nearly all studies to date, increased expression of the induced-Environmental Stress Response (iESR) genes is coupled to reduced expression of RP and RiBi genes in the repressed-Environmental Stress Response (rESR) gene set. Whether regulation of the iESR and rESR genes can be decoupled in wild-type cells is unclear.

Msn2 overexpression is sufficient to induce multistress tolerance in yeast cells [39]. Thus, cell-to-cell variation in Msn2 activation could explain the heterogeneity in stress tolerance in an actively growing culture. But it remains unclear if this variation correlates with broader transcriptome changes, if the magnitude of the response in unstressed cells mimics that seen in stressed cells, or if fluctuations in the response correlate with cell-cycle phase. Here, we addressed these questions through single-cell RNA-sequencing (scRNA-seq) coupled with single-cell profiling of transcription factor activation dynamics before and after stress. Our results reveal variable activation of both the ESR and condition-specific regulators after stress and heterogeneity in ESR activation in unstressed cells due to both coordinated and discordant induction of ESR regulators. While ESR activation shows no relation to cell-cycle phase in unstressed cultures, we found that some cells appear to decouple regulation of RP transcripts in a manner linked to S-phase but apparently unrelated to expression changes associated with the yeast metabolic cycle.

## Results

We used the Fluidigm C1 system to perform scRNA-seq on actively growing yeast cells collected from rich medium, before and 30 min after treatment with 0.7 M sodium chloride (NaCl) as a model stressor. Although the Fluidigm system generally profiles fewer cells than other methods, it has a substantially higher capture rate enabling deeper investigation of the cellular transcriptome [40,41]. Cells were immediately fixed by flash freezing to preserve the transcriptome during the capture process. We optimized a protocol to capture partially spheroplasted yeast cells on the C1 platform, ensuring that cells remained intact during capture but lysed in the instrument. We performed two C1 chip runs for each of the unstressed and stressed cultures and selected 85 and 81 captured single-cells, respectively; 83 and 80 yielded successful libraries (see Methods). Libraries were pooled, paired-end fragments were sequenced, and identical fragments were collapsed to a single count to minimize amplification biases, producing a median of 1.4 million mapped de-duplicated fragments per cell (see Methods). Each transcriptome encompassed 735–5,437 mRNAs (median = 2,351), with a total of 5,667 out of the 5,888 yeast transcripts [42] covered by at least 5 reads in ≥ 5% of cells (S1 Table). As is well known for scRNA-seq, low-abundance transcripts with fewer read counts displayed a lower detection rate (i.e., lower fraction of cells in which the transcript was measured), likely a result of both technical noise and true biological variation [41,43–45]. The averaged responses of all stressed cells compared to all unstressed cells agreed well with bulk measurements (the correlation between log_2_(fold change) is 0.7), especially for stress-regulated genes (see S4 Fig), validating our procedure. For much of our analysis, we focused on the log_2_(normalized read counts) for each transcript in each cell, scaled to the mean log_2_(normalized read counts) of that mRNA in all other cells in the analysis—we refer to this as “mean-centered log_2_(read counts)” or “relative log_2_ abundance.” Thus, positive log_2_ values indicate expression above the population mean of that transcript, and negative values represent expression below the mean.

### Quantitative variation in ESR expression

**Fig 1:**
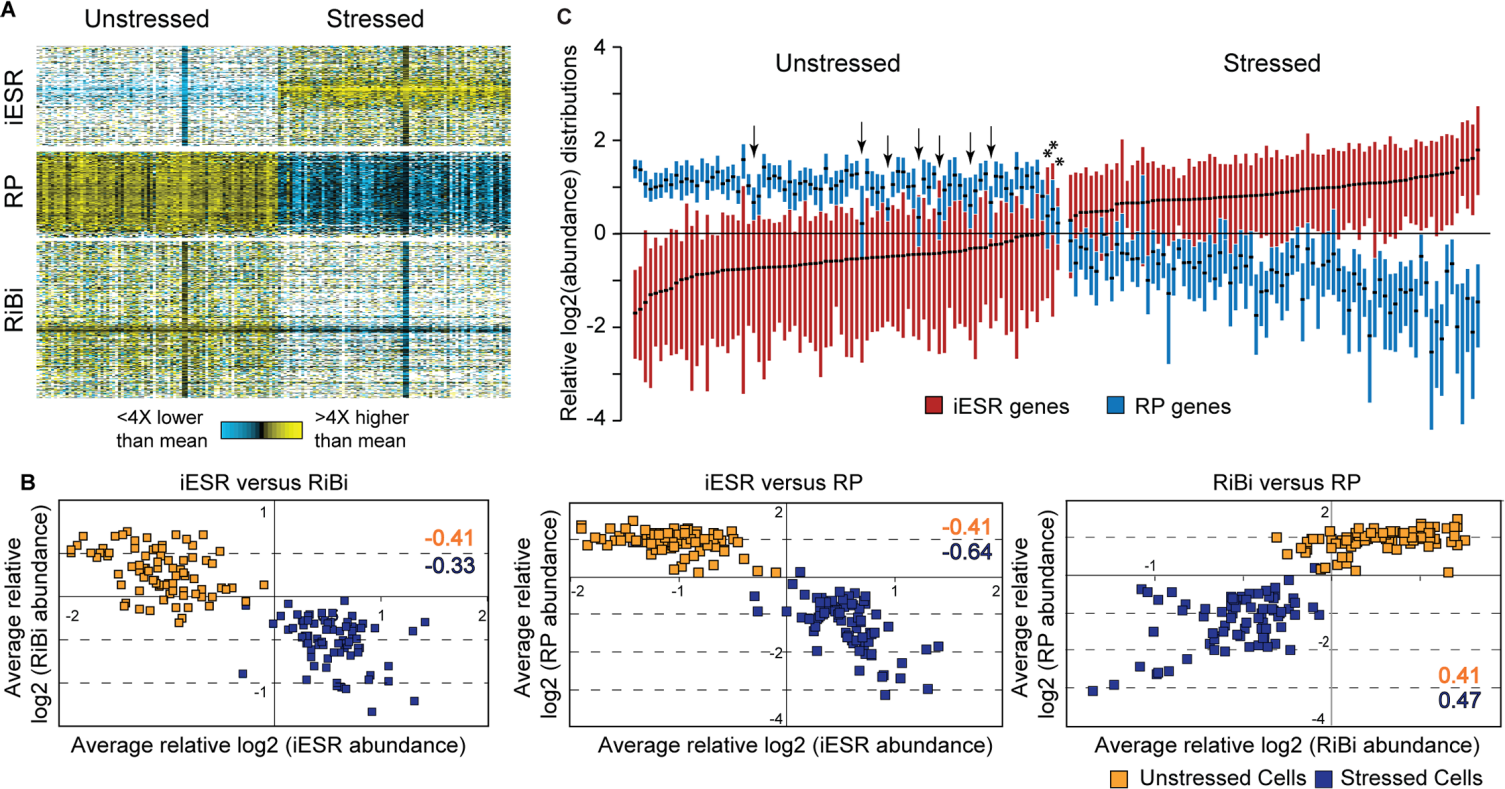
Quantitative variation in ESR activation across cells. (A) Mean-centered log_2_(read counts) for ESR gene groups before and after stress. Each row represents a transcript and each column is an individual cell, with expression values according to the key; white indicates no detected transcript. (B) The average mean-centered log_2_ values for a given ESR gene group as measured in one cell was plotted against the average mean-centered log_2_ values for a second ESR gene group as measured in the same cell. Correlations for unstressed (orange) and stressed (purple) cells are indicated on each plot. (C) Boxplots (without whiskers) of mean-centered log_2_(read counts) of RP and iESR transcripts in individual cells, sorted by iESR-group median. Arrows indicate unstressed cells with unusually low RP transcript abundances (FDR < 0.05, see Quantitative variation in ESR expression) and asterisks indicate those cells that also had high median iESR log_2_ values. ESR, Environmental Stress Response; FDR, false discovery rate; iESR, induced-Environmental Stress Response; RiBi, ribosome biogenesis; RP, ribosomal protein.

As expected, stressed and unstressed cells could be readily distinguished based on their cellular transcriptome, primarily driven by expression of the ESR genes (Fig 1A). Most unstressed cells displayed relatively high abundances of RP and RiBi transcripts and low abundances of iESR transcripts, consistent with ESR suppression, whereas stressed cells displayed the opposite patterns, indicative of ESR activation. However, there was considerable variation in the magnitude of ESR activation, both before and after stress. Some stressed cells showed concertedly stronger activation of the ESR than other cells (Fig 1B and 1C). Among unstressed cells, at least 4% showed mild ESR activation, as evidenced by low RP expression relative to other unstressed cells (false discovery rate [FDR] < 0.05, *t* test, see Methods) coupled with high relative iESR mRNA abundances (Fig 1C, asterisks). In general, quantitative differences in ESR activation were correlated across ESR subgroups: cells with higher relative iESR transcript abundance generally showed lower relative RiBi and RP mRNA levels, whereas quantitative differences in RP abundance were generally correlated with RiBi abundances, especially in stressed cells (Fig 1B). The quantitative and correlated variation across these groups suggests coordinated cell-to-cell variation in ESR activation levels, both before and after stress.

But there was also evidence of group-specific variation that appeared decoupled from ESR activation. Of the 12% of unstressed cells with lower relative RP abundance (FDR < 0.05, *t* test), two-thirds did not show significant iESR activation or RiBi repression (Fig 1C arrows and S1 Fig). Likewise, a subset of stressed cells with strong iESR induction had significantly weaker repression (i.e., higher relative abundance) of RP transcripts (FDR < 0.05, *t* test, see more below). These results suggest that heterogeneity in other cellular covariates may influence expression of RP transcripts separate from full ESR activation, investigated in more detail below.

### Low variation in RP transcripts reflects tight cellular control

**Fig 2:**
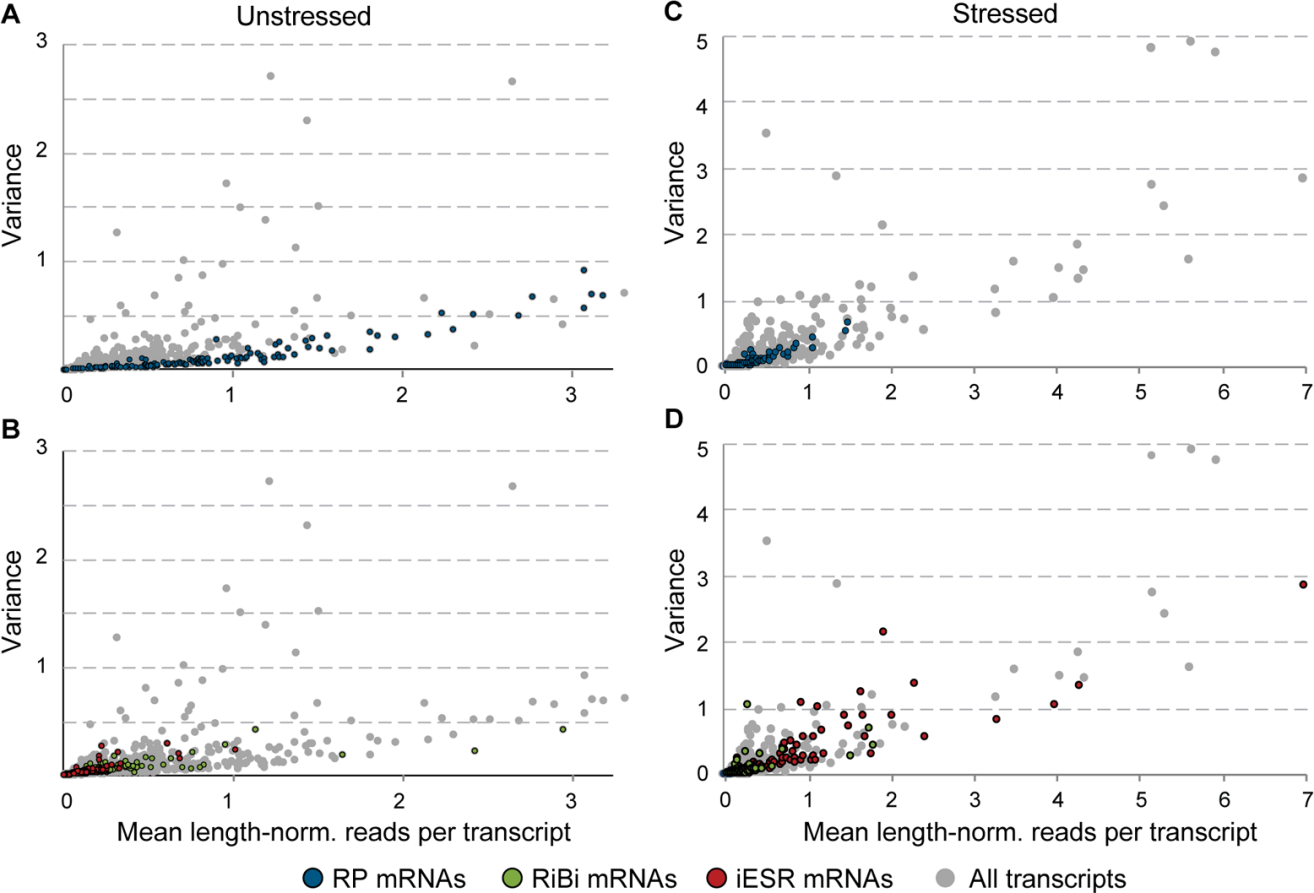
RP transcripts show low variation in abundance across cells. The mean and variance of transcript read counts per mRNA length (“length-norm”) was plotted for each mRNA from unstressed (left) or stressed (right) cells. (A,C) highlight RP transcripts and (B,D) highlight iESR and RiBi transcript against all other mRNAs (grey points). Plots are zoomed to capture most points. iESR, induced-Environmental Stress Response; RiBi, ribosome biogenesis; RP, ribosomal protein.

**Fig 3:**
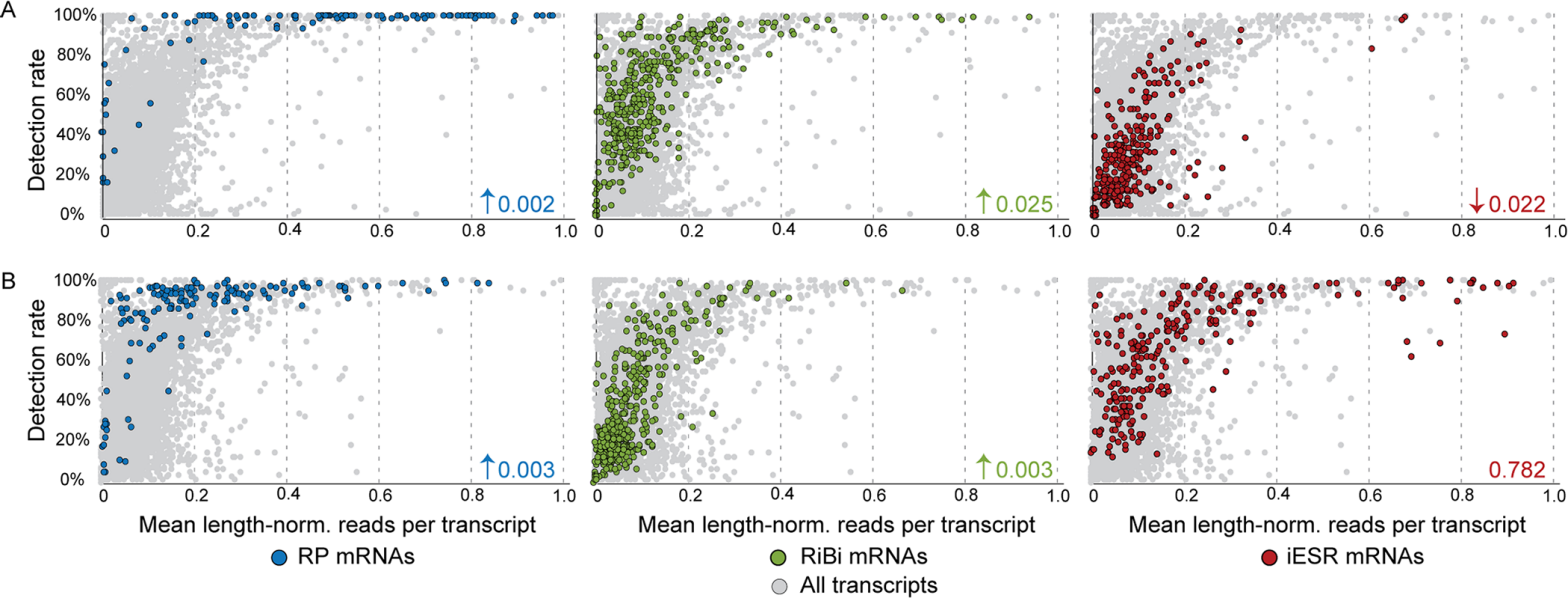
Transcript detection rate correlates with functional class. The fraction of cells in which each mRNA was detected was plotted against the mean length-normalized read count for that transcript, calculated from cells in which the transcript was measured, in (A) unstressed or (B) stressed cells. Listed *p*-values and arrows (where significant) indicate if the detection rate was higher or lower than randomly sampled genes. Plots are zoomed in to show transcripts whose mean read count is below 1.0; most transcripts above this range are detected in all cells, not shown. iESR, induced-Environmental Stress Response; RiBi, ribosome biogenesis; RP, ribosomal protein.

**Fig 4:**
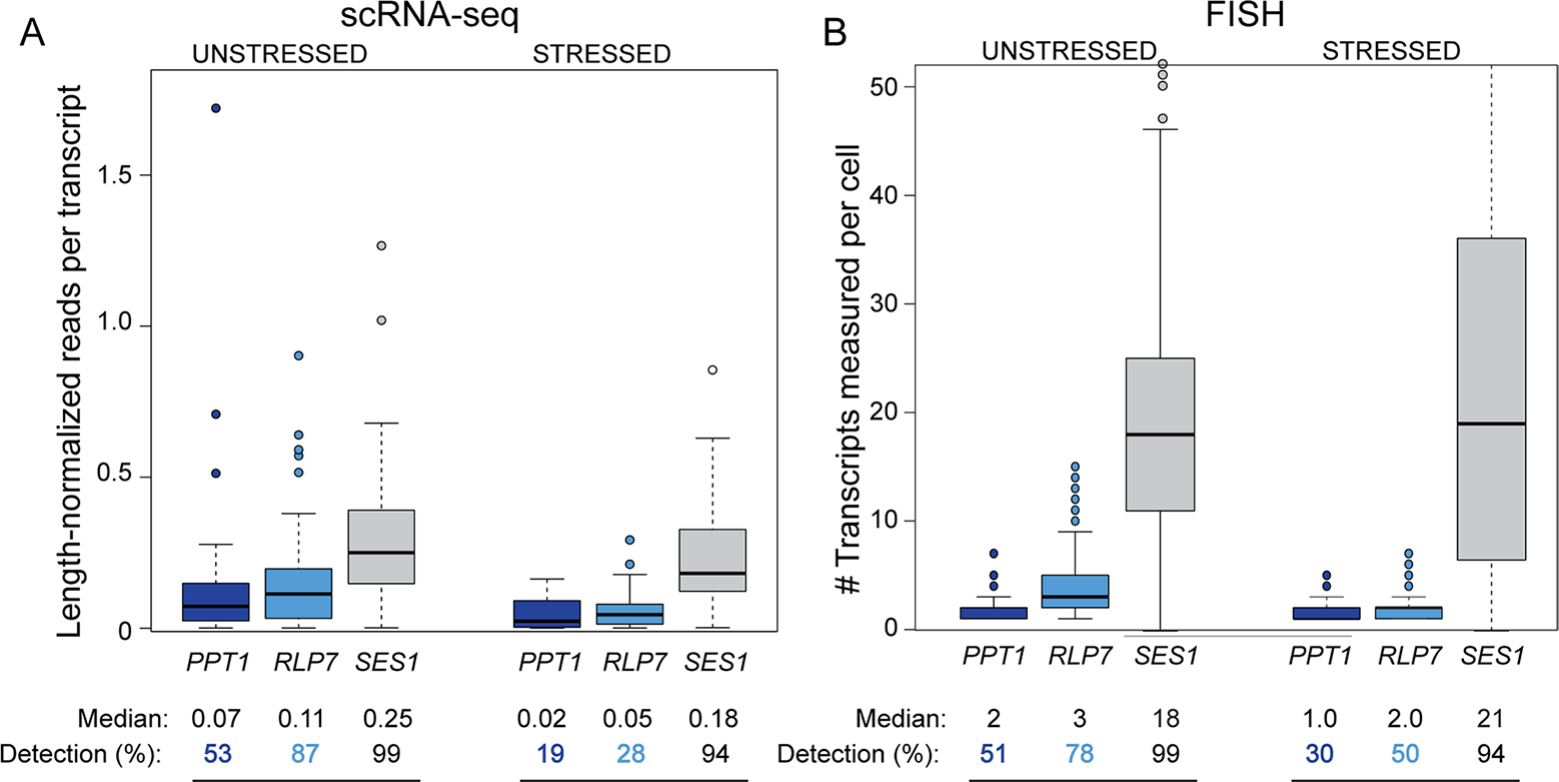
Single-molecule FISH confirms differences in detection rate at several transcripts. Distributions of (A) length-normalized read counts measured by scRNA-seq and (B) mRNA molecules per cell measured by single-molecule FISH, for *PPT1*, *RLP7*, and *SES1* as a control. Note only part of the *SES1* distribution is shown. Median counts in cells with a measurement and detection rate (percentage of cells with a measurement) are listed below the figure. Data are available in S10 Table. scRNA-seq, single-cell RNA sequencing.

In the process of this analysis, we noticed that RP mRNAs showed tight distributions with relatively low variance in unstressed cells (Fig 1B and 1C). In fact, RP transcripts showed among the lowest variances across the range of transcript abundances (Fig 2, *p* = 2e-59, see Methods), consistent with what has been reported at the protein level [19]. The distinction persisted in stressed cells, although RP variance was notably higher after stress treatment (Fig 2C, *p* = 3e-22). We also noticed that the detection rate for RP transcripts appeared to be exceedingly high, even for RP transcripts known to be expressed at low abundance [46]. To investigate this, we plotted the detection rate versus read counts per transcript length as a proxy for mRNA abundance and devised a statistical test based on cubic splines to identify differences across gene groups (see Methods). As a group, RP transcripts (*p* < 0.0028) and, to a lesser extent, RiBi mRNAs (*p* < 0.025) showed significantly higher detection rates for their abundance levels compared to randomly chosen genes for both stressed and unstressed cells (Fig 3). The result was not an artifact of known covariates of RP mRNAs. Although RP mRNAs are generally short, they remained statistically different from randomly chosen transcripts that are shorter than the median RP length (*p* < 0.0012). Many RPs also have close paralogs in the genome, which could obscure true abundance if reads mapping to multiple locations are discounted from the alignment. But RPs remained significant, at least for unstressed cells (*p* = 0.02), when the analysis was performed only on mRNAs without a close paralog (Basic Local Alignment Search Tool [BLAST] E value > 1e − 5). We validated the difference in detection rate for several transcripts in the same abundance range using single-molecule mRNA fluorescence in situ hybridization (sm-FISH, Fig 4). *RLP7*, encoding a ribosome-associated RP-like protein, and phosphatase-encoding *PPT1* transcripts were sequenced to similar read densities but were detected in 87% versus 53% of unstressed cells, respectively. The distinction was confirmed by sm-FISH: *RLP7* was measured in 78% and 50% of cells collected before and after stress, respectively, whereas *PPT1* was measured in 50% and 30% of cells, respectively, despite similar abundance ranges when present.

One likely reason for the tight control on RP transcripts is the importance of stoichiometric protein expression for proper ribosome assembly, and several RP transcripts are subject to extensive regulation to impose this control [47–50]. We sought other transcripts whose detection rate was higher than predicted by their abundance and identified mRNAs (lacking paralogs) that were above the RP-fit spline (S2 Table). Remarkably, this group was heavily enriched for mRNAs encoding multisubunit protein complexes [51] in the analysis of both unstressed (*p* = 3.5e-4) and stressed (*p* = 2e-25, hypergeometric test) cells. This included subunits of the proteasome, chaperonin-containing T complex, and nuclear pore, as well as mRNAs encoding proteins destined for various subcellular regions, consistent with past sm-FISH analysis [52]. We also found mRNAs at the opposite end of the spectrum: iESR mRNAs showed 15% lower median CV (coefficient of variance) than other transcripts but only after stress (*p* = 8e-7, see Methods). By contrast, the iESR mRNAs from unstressed cells displayed slightly higher variance (*p* = 6e-5) as previously reported at the protein level [19] and lower-than-expected detection rates (*p* = 0.022). Many iESR genes are regulated by burst-prone TATA-containing promoters [13,15–17], and indeed, other TATA-regulated genes without paralogs were weakly enriched among those with unusually low detection rates (*p* = 0.01). Together, these results confirm that the detection rate is not merely a function of technical variation [41,43] and show that transcripts in different functional groups are subject to different regulatory constraints per cell but that these constraints can vary by environment [19,53].

### ESR activation does not fluctuate with cell-cycle phase

**Fig 5:**
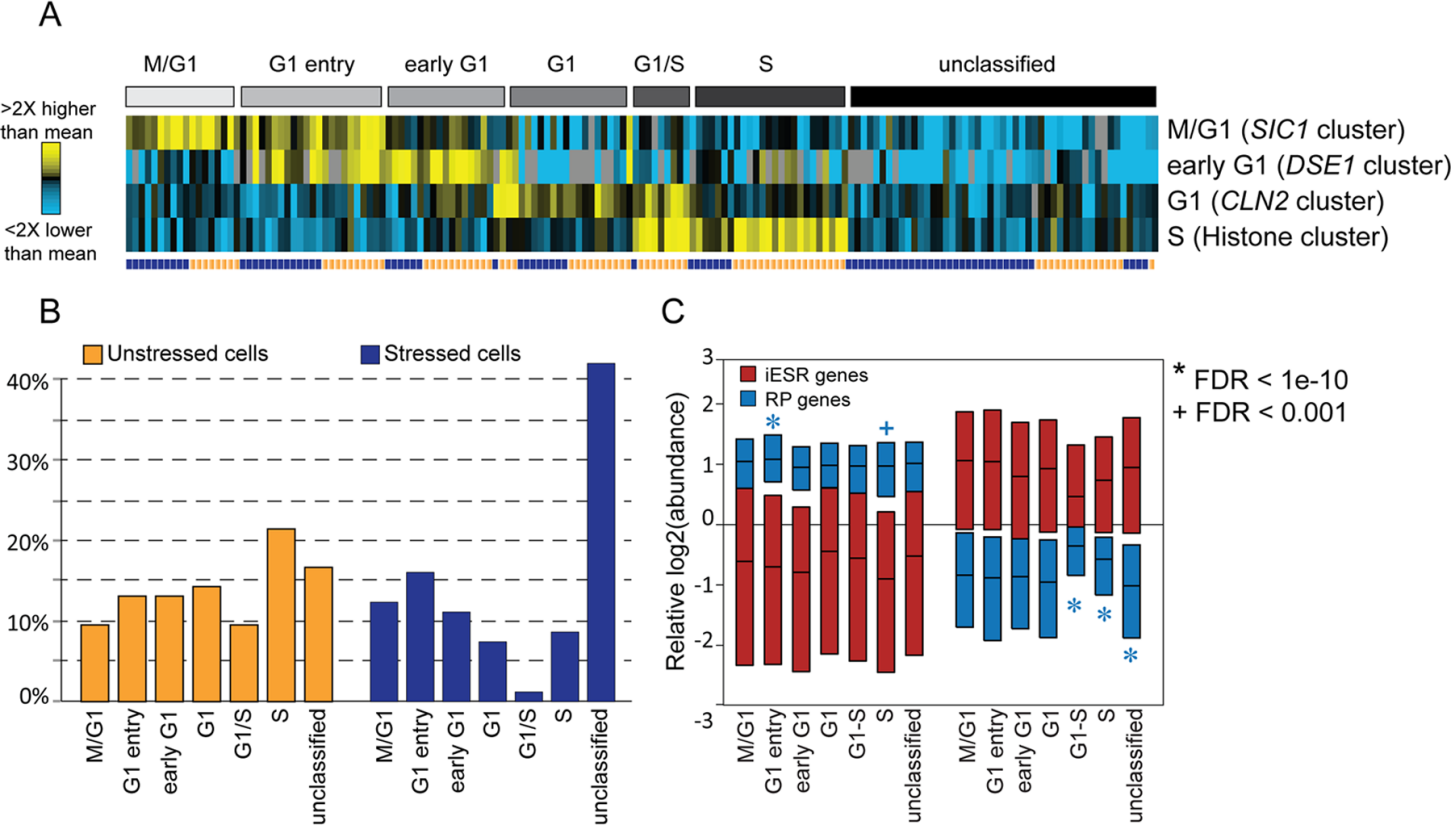
The influence of cell-cycle phase on ESR activation. Cycling genes used for classification were identified by clustering the scRNA-seq data [54] and then selecting clusters enriched for cell-cycle markers (S3 Table, see Methods). (A) Cells (columns) were clustered based on the centroid expression pattern of genes within each group (rows) and manually classified into and sorted within designated groups (A, grey bins, S9 Table). Stressed and unstressed cells are annotated by the purple/orange vector (A, bottom row). (B) The percentage of cells in each cell-cycle phase. Cell phases are listed in S5 Table. (C) Boxplots (without whiskers) of all iESR (red) or RP (blue) genes from cells in that phase. Significance was assessed by Welch *t* test on the pooled RP or iESR genes from cells within a given phase compared to all other cells; unstressed and stressed cells were analyzed separately. Note only one cell was classified as G1/S after stress. ESR, Environmental Stress Response; iESR, induced-Environmental Stress Response; RP, ribosomal protein; scRNA-seq, single-cell RNA sequencing.

The analysis in Fig 1 revealed variation in ESR activation across cells, both before and after stress, as well as decoupling of RP expression in some cells. We hypothesized that this variation could emerge if cells were in different physiological states. The first candidate was cell-cycle phase. We used the program Pagoda [54] to identify clusters strongly enriched for known cell-cycle mRNAs and then classified cells based on the expression peaks of the cluster centroids (Fig 5A, S3 Table, see Methods). A subset of cells could not be classified, in part because we were unable to identify coherent expression among mitosis-phase (M-phase) genes. Comparing the fraction of cells in each phase before and after stress recapitulated the known G1 delay after osmotic stress [55,56] (Fig 5B). Interestingly, many more cells could not be classified based on their expression profile after NaCl treatment; while some of these cells could be in G2/M phase, our results are consistent with the notion that the transcriptome of arrested cells may not necessarily mimic that of cycling cells [57,58].

We found no evidence that ESR activation as a whole is a function of the cell cycle. There were no statistically significant differences in the coordinate expression of iESR, RP, and RiBi transcripts at different points in the division cycle, including G1 phase. The only trend across the collective ESR groups was seen in stressed cells progressing through S-phase at the time of collection: these cells showed significantly higher abundance of RP transcripts (*p* < 1e-10) and, although not significant, slightly higher abundance of RiBi mRNAs and lower abundance of iESR transcripts (S2 Fig). The simplest explanation is that these cells have partly acclimated and are thus relaxing ESR activation as they re-enter the cell cycle after G1 delay triggered by NaCl treatment [55,56].

Although ESR activation as a whole was not coupled to cell-cycle phase in unstressed cells, we were surprised to find concerted differences in RP expression, separable from activation of the ESR. Cells in early G1 had slightly, but statistically significantly, higher expression of RP transcripts; this phase represents the period of maximal cell growth in yeast. In contrast, a third of unstressed cells in S-phase displayed concertedly low expression of RP mRNAs (FDR < 0.05, S2 Fig)—these accounted for many of the cells identified in Fig 1C. The reduced expression of RP mRNAs was not related to higher iESR abundance or lower RiBi mRNA levels, aside from three cells in which the ESR appeared to be weakly activated (S1 Fig). Thus, RP expression appears to be decoupled from ESR activation in a subset of unstressed cells.

### No evidence for mRNA cycling in the yeast metabolic cycle program

One potential link between RP expression and S-phase is the ultradian yeast metabolic cycle (YMC), which can be synchronized in bulk cultures through nutrient deprivation and has recently been reported in asynchronous, nutrient-replete cultures [59–62]. In starvation-synchronized cultures, bulk transcriptome analysis identified three YMC phases, including an oxidative phase in which RP mRNAs peak, a reductive building phase in which respiration factors peak, and a reductive charging phase in which transcripts involved in fatty acid metabolism and glycolysis are maximal [59]. The reductive building phase is at least partly aligned with S-phase of the cell cycle [59,60,62,63], which could explain why a subset of S-phase cells display low RP expression.

However, we did not find evidence for the same YMC transcriptome program reported in nutrient-restricted chemostats. First, there was no evidence that RP transcripts are cycling in our dataset. We used the program Oscope [64] to identify cycling transcripts, which were heavily enriched for cell cycle-regulated mRNAs (*p* = 2e-16, hypergeometric test [65]) but not RPs or transcripts encoding metabolic enzymes (S4 Table). Second, we sought other RNAs whose patterns varied in accordance with RPs. Unstressed cells were ordered based on five representative RP transcripts using the WaveCrest algorithm [66], which then identified other mRNAs whose profiles fluctuated according to the same cell ordering (but not necessarily the same abundance profile, see Methods). Out of the top 100-ranked transcripts, most were RPs or mRNAs encoding translation factors (S5 Table); one (*ENO1*) encoded a glycolysis enzyme, and several were localized to mitochondria, but there was no enrichment for these categories. Finally, we looked explicitly at the relative abundances of RP, glycolysis, and other YMC mRNAs (S3 Fig). There was cell-to-cell variation in abundance of glycolytic mRNAs consistent with the YMC expectation; however, there was no statistically significant link to RP abundance. Furthermore, there was no evidence that other transcripts associated with the YMC oscillated in our study, either in abundance or detection rate within cells (S3 Fig). Together, our results suggest that cells growing in rich medium may not display the same type of YMC-related transcriptome program as seen clearly in slow-growing nutrient-restricted cells [59,60].

### Heterogeneity in transcription factor targets implicates variation in regulation

We next searched for evidence of other cellular states that might influence expression of ESR gene groups. We leveraged the extensive knowledge of *S*. *cerevisiae* transcription factor (TF) targets to explore regulatory variation in single cells by identifying cells with concerted expression differences in sets of TF targets in two ways. First, we applied a gene-set enrichment approach to identify TF targets enriched among the distribution tails of relative log_2_ abundances in each cell (see Methods). Second, we applied a *t* test per cell, comparing relative abundance of each group of TF targets to relative abundance of all other transcripts in that cell—although the latter approach may lack statistical power, it is sufficient to detect strong skews in TF-target behavior.

**Fig 6:**
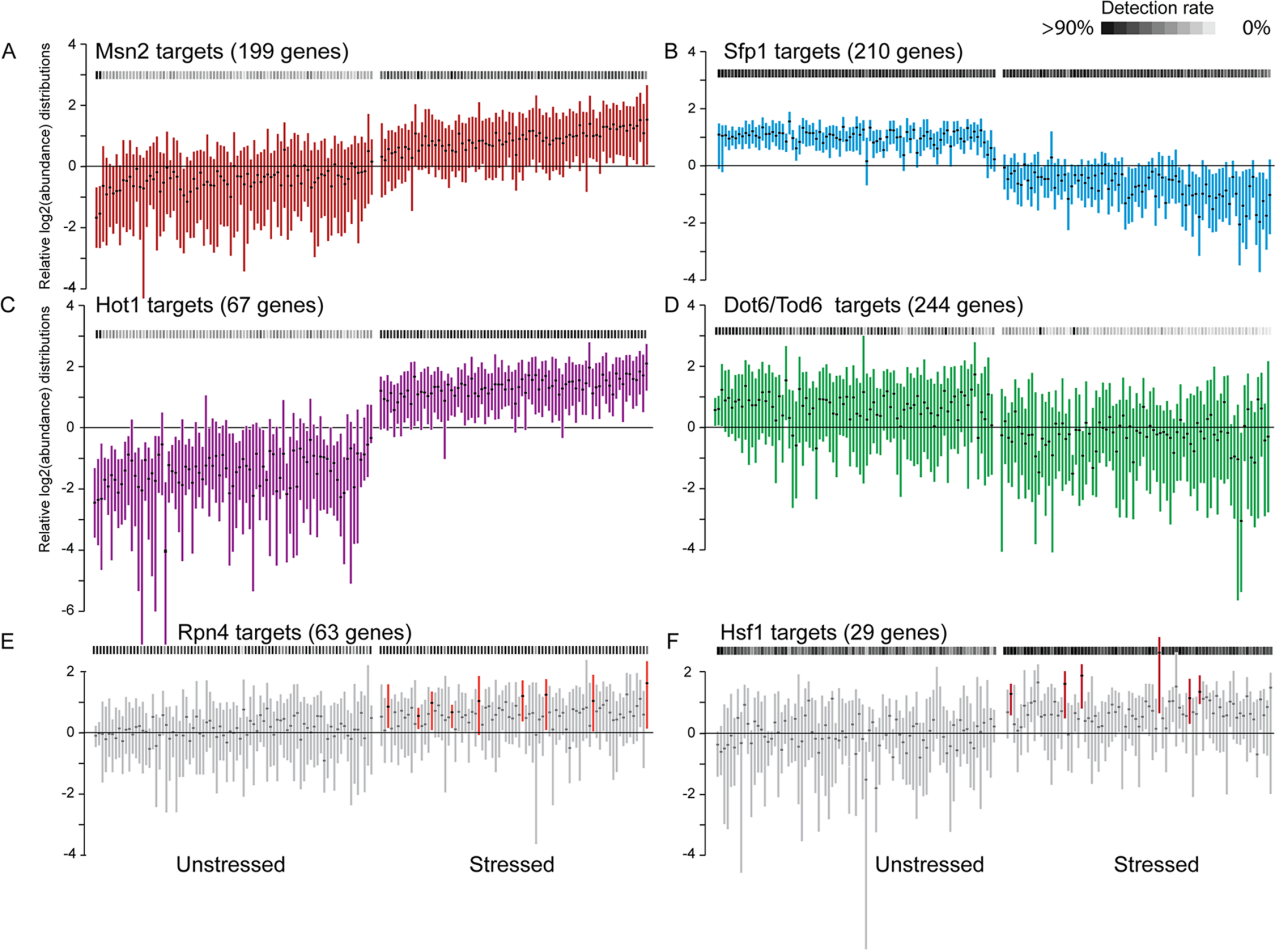
Regulatory variation across single cells. Distribution (without whiskers) of mean-centered log_2_(read count) values for indicated TF targets in single cells, organized as in Fig 1C. The number of targets for each TF is shown in parentheses. Grey-scale heat map (horizontal boxes) represents the detection rate, according to the key. (A-D) Targets of TFs that were differentially expressed in a large fraction of cells (see S6 Table). (E-F) Cells for which targets of Rpn4 (E) or Hsf1 (F) were significantly elevated (FDR < 0.053) compared to all other stressed cells are colored. TF, transcription factor.

These approaches identified eight sets of TFs whose targets were coherently differently expressed in at least 3% of cells (S6 Table). Several TF targets were differentially expressed in a large fraction of cells (Fig 6), including those of RP regulators (Ifh1/Fhl1, Sfp1, and the multifunctional Rap1 [67,68]), Dot6/Tod6 that repress a subset of RiBi genes during stress [69], and stress-responsive activators (Msn2, Hot1, Sko1 that regulate an overlapping set of targets). Several other factors were only implicated in a subset of cells, including cell-cycle regulators, as expected, but also proteasome regulator Rpn4 and heat-shock transcription factor Hsf1. In bulk RNA sequencing (RNA-seq) experiments, proteasome genes appear weakly induced by NaCl (S4 Fig)–our results instead show that Rpn4 is much more strongly activated in 11% of cells (FDR < 0.05), an effect that is lost in culture-level analysis. Hsf1 is not known to be activated by NaCl, and its targets are not coherently induced in bulk RNA-seq experiments (S4 Fig). Yet we identified higher expression of Hsf1 targets in 8% of stressed cells (FDR < 0.053), independent of Rpn4 target abundance. Thus, cells experience variation in signals related to protein degradation and folding in response to NaCl.

### Variation in TF relocalization reveals intrinsic and extrinsic variation in ESR regulation

To investigate the regulatory underpinnings of ESR variation revealed by scRNA-seq, we used single-cell microscopy to trace activation of the regulators implicated above. Cytosolic Msn2 and Msn4 rapidly relocalize to the nucleus upon various stress treatments; the same is likely true for Dot6 and Tod6 [29,30,70–72]. In contrast, the rESR activator Sfp1 is nuclear during active growth but ejected from the nucleus (and in some cases degraded) during stress to decrease RP transcription [27,28,73]. Several upstream regulators also change localization during stress, notably the NaCl-activated Hog1 mitogen-activated protein kinase [74]. While single-cell variation in Msn2/4 and Hog1 relocalization have been individually quantified [75–80], whether nucleocytoplasmic shuttling of these regulators before stress is coupled or fluctuates independently due to stochastic noise is not known. Furthermore, heterogeneity and dynamics of Dot6 and Sfp1 have not been investigated.

**Fig 7:**
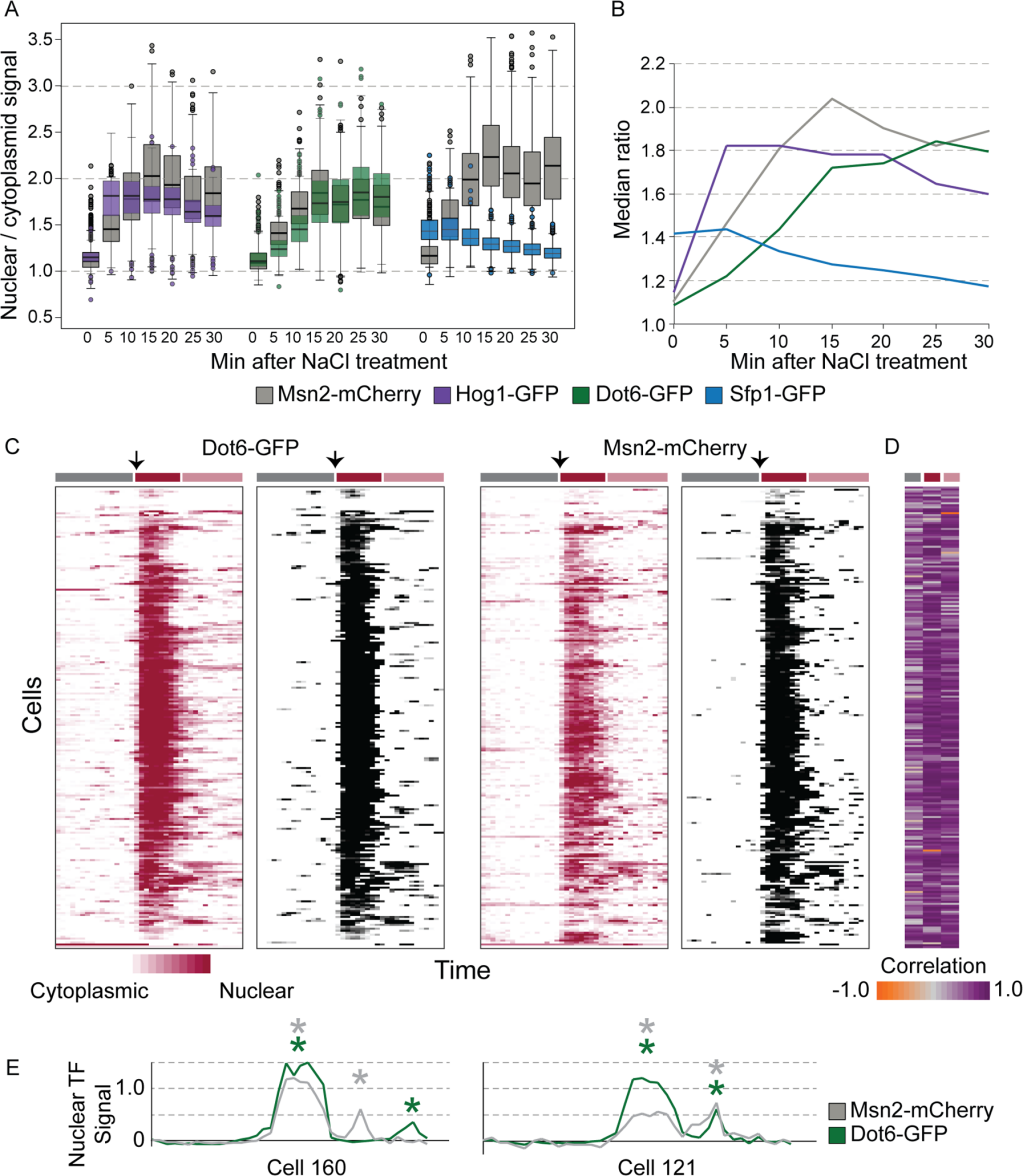
Stress-activated regulators show both coordinated and decoupled nuclear localization. (A) Distribution of nuclear/cytoplasmic signal for paired factors in individual cells before and after NaCl treatment (average *n* = 676 cells per time point). Data from two biological replicates were very similar and combined (S11 Table). (B) Median ratios from (A) plotted over time; the Msn2 plot combines measurements from all three strains. (C) Nuclear TF signals (see Methods) of Dot6-GFP (left) and Msn2-mCherry (right) expressed in the same cells over time, before stress and after NaCl addition at 81 min (arrows). Each row aligned across all plots represents a different cell, and each column represents a different time point. Red plots show traces of nuclear localization according to the key (see Methods), and corresponding grey-scale plots show quantitative measurements only for time points called as peaks. Colored boxes above the plots indicate 80 min before stress (grey box), 30 min after NaCl treatment (dark red box), and beyond 30 min after NaCl treatment (pink box). Data are available in S12 Table. (D) Correlation between Dot6-GFP and Msn2-mCherry traces for each temporal phase, according to the key. (E) Representative traces from (C), where called peaks (colored according to key) are indicated with asterisks. TF, transcription factor.

We therefore followed Msn2-mCherry localization in cells that also expressed Hog1, Dot6, or Sfp1 fused to green fluorescent protein (GFP). We first quantified dual-factor localization in fixed cells. No cells showed nuclear Hog1 before stress, but 12% and 10% showed nuclear Msn2 or Dot6, respectively (based on identifiable nuclear objects, see Methods), consistent with the stressed state. However, only a third of cells with one factor localized to the nucleus also showed nuclear localization of the other. A small fraction (approximately 4%) of unstressed cells showed a dearth of nuclear Sfp1 signal (below the median ratio seen 30 min after stress, Fig 7A)—but there was no evidence of nuclear Msn2 in any of these cells. Upon NaCl treatment, the factors showed distinct dynamic behavior, with nuclear Hog1 peaking at 5 min, followed by maximal Msn2 and Dot6 nuclear localization at 15 min and 25 min, respectively (Fig 7B). Relocalization of Sfp1 was significantly prolonged and had not plateaued by 30 min after stress, consistent with the timing of transient rESR transcript reduction which troughs at 30–45 min after NaCl treatment [31].

We were especially interested in potential decoupling of ESR TFs, particularly in unstressed cells. However, differences in relocalization dynamics confound the analysis, since it could mimic decoupling in single-timepoint snapshots. We therefore followed TF dynamics in living cells, quantifying TF localization (see Methods) every 300 sec (to minimize light-induced stress [75]) and calling temporal peaks or troughs in nuclear concentration (see Methods). We were unable to confidently call troughs of nuclear Sfp1 before stress, but there appeared to be cells in which Sfp1 was depleted from the nucleus with no sign of nuclear Msn2 during the 80-min unstressed time course (S1 Data). 19% and 22% of cells showed a detectible peak of nuclear Dot6-GFP or Msn2-mCherry, respectively, during 80 min of unstressed growth; but only 8% of cells showed nuclear translocation of both factors at some point during the experiment, generally with similar timing (median correlation in traces = 0.55, Fig 7C and 7D, S2 Data). This fraction is higher than the joint probability of independent regulation (4%) and in close agreement with the 4% of cells for which scRNA-seq implicated weak ESR activation. Nonetheless, there were clear cases of decoupling (Fig 7E): over a third of unstressed cells with a Dot6–GFP nuclear transition showed no called peak in nuclear Msn2–mCherry and low correlation (< 0.2) in unstressed traces. These cells do carry unmarked Msn4, but it rarely transits to the nucleus in the absence of added stress and upon stress treatments generally correlates closely with Msn2 [72]. In all cases studied here, pre-stress nuclear pulses were both shorter and milder quantitatively compared to after-NaCl treatment. Both factors transited to the nucleus upon NaCl treatment in almost all cells, after which time cells showed nucleocytoplasmic bursts that were partly decoupled (across cells and factors, see Fig 7E), as previously reported for Msn2 [81]. Thus, while Dot6 and Msn2 activation were highly correlated during the acute response to NaCl, the pre-stress and post-acclimation phases showed evidence of both coordinated and decoupled nuclear fluctuations of the regulators.

## Discussion

Our work addresses several unanswered questions regarding heterogeneity in stress defense and tolerance. Many past studies have characterized the transcriptomic responses to stress at the culture level, presenting a wealth of information on the modes and mechanisms of stress defense. Other studies have characterized variation in protein abundance across individual yeast cells, but generally only one or two proteins at a time [19–22,82]. A critical missing component from past studies is how and why individual cells vary in their cellular response. Our results indicate that individual yeast cells can vary substantially in the magnitude of their transcriptome response, both before and after stress, and that individual cells experience stress differently (exemplified by quantitative differences in ESR activation and differential expression of proteasome- and chaperone-encoding transcripts after NaCl treatment). The extensive knowledge of yeast transcriptional regulation enabled us to investigate sources of upstream transcriptome regulation, implicating heterogeneity both intrinsic to individual regulatory paths and extrinsic to the cellular system.

### Heterogeneous ESR activation before stress likely influences stress survival

Both the scRNA-seq results and fluorescent TF profiling suggest that a subset of cells mediate mild activation of the ESR in the absence of added stress. Four percent of cells showed mildly higher iESR and lower RP mRNA abundance (Fig 1C), consistent with the 4% of cells estimated to coregulate iESR/rESR regulators Msn2 and Dot6 (Fig 7C). We propose that this mild activation contributes to the heterogeneity in single-cell survival of extreme stress doses. Although we observed decoupled Msn2 and Dot6 pre-stress nuclear fluctuations by microscopy, we did not observe decoupled activation of their combined targets in individual unstressed cells (Fig 1). The amplitude and duration of pre-stress Msn2/Dot6 pulses were significantly smaller and shorter than immediately after NaCl stress. In the case of Msn2, relocalization is influenced by both nuclear import and export that together produce distinct temporal profiles [76,83–85]. One prediction is that genes with more Msn2 binding sites are more sensitive to brief pulses of nuclear Msn2 [23,86,87]. However, our data did not support this: genes with many Msn2 binding sites showed no more evidence of concerted pre-stress fluctuations than genes with few binding sites (S5 Fig). This suggests that cells maintain a filtering system to distinguish a true upstream signal from noisy TF activation. This system could emerge from chromatin regulation [86,88] or other regulatory signals (e.g., post-translational TF modification) that act as gatekeepers to the transcriptome response.

A remaining question is why some unstressed cells activate the ESR program. One model is that stochastic fluctuations in a common upstream regulator produce stochastic but coordinated activation of the downstream factors. A candidate is PKA, which phosphorylates and suppresses several stress-activated regulators (including Msn2 and Dot6), promotes expression of RP transcription [89,90], and has been implicated in stochastic Msn2 regulation [22,70,85,91]. Whether PKA fluctuations represent random events or a response to some cellular signal is not clear. A second, compatible model is that cells with mild ESR activation are actually experiencing, and thus actively responding to, internal stress. Such stress could emerge from normal cellular processes, e.g., damage from bursts of oxidative metabolism or during DNA replication. A third model that our data discounts is that the ESR fluctuates with the cell cycle in normally dividing cells [38]. We did see a milder ESR activation in post-stress cells in S-phase, but we believe this is due to acclimation-dependent re-entry into the cell cycle. We propose that the previously reported correlation between ESR activation and prolonged G1 in mutants is likely a response to deleterious gene deletions rather than an inherent coupling of the ESR to G1 phase.

### Exquisite control of RP transcripts can be decoupled from the ESR

In many bulk transcriptomic yeast studies to date, RP expression is inversely proportional to stress-defense transcripts in the iESR, and these gene groups display opposing responses during rapid growth versus environmental stress [24,37,39]. Indeed, these gene groups are controlled by the same upstream signaling pathways, including PKA, TOR, and stress-activated regulators [92–94]. But studying individual cells expands knowledge of the regulatory system: although RP and iESR transcripts are anticorrelated in most cells in our analysis, RP expression appears decoupled in a subset of unstressed individuals. The reason and mechanisms remain unclear. The link between low RP transcripts and S-phase is appealing: although we found no clear evidence for the same YMC transcriptome seen in nutrient-synchronized cultures [59], metabolic genes are regulated during the G1/S transition [95,96]. Furthermore, the yeast cyclin dependent kinase, Cdc28, also binds to RP promoters [97], and an imbalance of ribosome components can trigger G1/S delay [98,99]. Future work will be required to decipher this regulation, as well as the mechanisms that give rise to exquisite control minimizing variation and ensuring cell presence of RP mRNAs.

### Implications for heterogeneous stress responses in other organisms

Heterogeneity in microbial stress tolerance has been proposed to serve as a bet-hedging mechanism, ensuring that a minimal fraction of the population survives in the event of catastrophic environmental events [6,7,10]. But the phenomenon is also observed in multicelled mammalian systems [2–4,100] and at least partly influenced by variable activation of the p53 tumor suppressor. Like Msn2, inactive p53 resides in the cytoplasm but upon stress rapidly relocates to the nucleus with transient pulses, where it activates target–gene transcription and has been reported to repress ribosome-producing polymerase I and III [94,101–103]. p53 gene targets that harbor multiple and high-affinity binding sites are most sensitive to transient nuclear bursts, as reported previously for Msn2 targets, whereas other genes require prolonged p53 activation for full induction [101,104,105]. And as is the case with Msn2 activation, prior induction of p53 leads to subsequent tolerance to what would otherwise be lethal drug doses [106]. p53 also shows heterogeneous nuclear pulses in proliferating cells without exogenous stress. Unlike Msn2, which shows quantitatively shorter and weaker pre-stress nuclear pulses, the amplitude and duration of pre-stress p53 pulses is reported to be similar to that seen after inflicted stress; but like the yeast factor, these pre-stress bursts do not necessarily alter gene expression [107]. Instead, layers of post-translational p53 modification can filter potential noise in the regulatory system. A better understanding of the regulatory networks that control heterogeneity in transcription and stress tolerance is likely to open new avenues to control population behavior.

Our results present new insights into heterogeneity in the yeast stress response, but many unanswered questions remain. One is how dynamic changes in TF activation relate to dynamic changes in transcript abundance and, in particular, how the frequency and amplitude of TF relocalization quantitatively impact mRNA output. Another is the extent to which cellular dynamics vary across cells, e.g., in terms of how variable cells are in their acclimation to stress. Single-molecule approaches in living single cells will be an important avenue to dissect these questions. In terms of physiology, it will be interesting to dissect how variations in cellular states and systems influence the transcriptome and stress responses more broadly—why do some unstressed cells have lower RP expression and other stressed cells activate unique TF responses? As the accuracy of scRNA-seq improves, so too will the ability to infer physiological differences in the cellular system that influence heterogeneity in stress tolerance and susceptibility.

## Methods

### Strains and growth conditions

All experiments were done in the BY4741 background. Unless noted, cells were grown in rich YPD medium in batch culture at 30 °C for at least seven generations to mid-log phase, at which point an aliquot was removed to serve as the unstressed sample. NaCl was added to a final concentration of 0.7 M in the remaining culture and cells were grown for 30 min. Unless otherwise noted for specific applications, cells were collected by brief centrifugation, decanted, and flash frozen in liquid nitrogen. Strains expressing tagged proteins were generated by integrating an mCherry-*HIS3* cassette downstream of *MSN2* in BY4741 strains from the GFP-tagged collection [108], which were verified to harbor the GFP–*HIS3* cassette downstream of *DOT6*, *SFP1*, or *HOG1* (generating strains AGY1328, AGY1329, and AGY1331, respectively).

### Single-cell sorting, library preparation, and sequencing

Fluidigm’s C1 microfluidic platform was adapted to perform cDNA synthesis from single yeast cells. Flash frozen cells were resuspended in 1 mL of 1 M Sorbitol on ice, counted on a hemocytometer, and then diluted to approximately 4 × 10^5^ cells per mL in a final volume of 200 μL. To generate partial spheroplasts that could easily lyse on the Fluidigm C1 microfluidic device, we titrated each sample with different amounts of zymolyase (0.025 U, 0.0125 U, 0.00625 U, and 0.003125 U) and incubated cells for 30 min at 37 °C. This was done because unstressed and stressed cells displayed different sensitivities to zymolyase digest. After incubation, cells were spun at 250 g for four min and resuspended in Sorbitol Wash buffer (0.455x C1 Cell Wash Buffer, 1 M sorbitol, 0.2 ug/μl BSA, 0.08(8) U/μL SUPERase RNAse Inhibitor). Samples with the maximal number of intact spheroplasts (compared on a Leica DMI 6000 inverted microscope) were diluted to a final concentration of 600 cells/μL, and 9 μL of these cells were mixed with Fluidigm Suspension reagent at final loading concentration of 275 cells/μL and loaded onto the primed C1 Chip designed to capture 5–10-μm cells, according to manufacturer instructions. The cell concentration in the loading mixture was crucial to maximize the number of wells capturing single cells inside the microfluidic device. Another modification was that 1 M Sorbitol was added to all wash buffers to prevent premature lysis. After cell loading, each chip was visually inspected and imaged to tabulate single-cell capture rates. Roughly 50% of wells contained a single cell, verified by imaging and manual inspection. This rate is lower than the normal capture rate because yeast cells are smaller and deform less. Spheroplasts were lysed in the Fluidigm instrument and cDNA was generated using Clontech reagents for Fluidigm C1 based on the single-cell RNA-seq protocol (cat # 635025). Finally, cDNA was harvested from each Fluidigm C1 chip and into a 96-well plate for storage at −20 °C. ERCC spike-in sequences (mix A) were added at 1:4 × 10^5^/μL of the concentration provided in the original product (Ambion catalog number 4456740).

Before library preparation, cDNA from each cell was quantified on an AATI Fragment Analyzer. Using concentrations calculated from a smear analysis between 450 bp and 4,500 bp, cDNA from each cell was diluted with TE to approximately 0.2 ng/μL using the Mosquito X1 pipetting robot (TTP Labtech). Diluted cDNA served as the template for Nextera XT library generation following manufacturer protocol (Illumina catalog number FC-131-1096) with some modifications. Because we used TTP’s Mosquito HTS 16 channel pipetting robot (capable of accurately aliquoting volumes down to 50 nL), we were able to scale down the total volume of each Nextera XT library to four μL. More specifically, for each 400 nL of input template DNA, we added 400 nL Tagmentation mix and 800 nL Tagmentation buffer for a final volume of 1.6 nL. The Tagmentation reaction was incubated at 55 °C for 10 min. Neutralization was done by adding 400 nL Neutralization buffer to the above reaction and incubating 10 min at room temperature followed by the addition of primers at 400 nL each and NPM PCR master mix at 1,200 nL. The PCR step was run for 10 cycles. One μL of each library was combined to form two separate pools, one for unstressed cells and one for stressed cells. Two rounds of size selection were performed using Agencourt AMPure beads (Beckman Coulter catalog number A63882). One hundred ng of each pool was combined and sequenced in one run on three lanes of an Illumina HiSeq-2500 1T v4 sequencer for 150-bp paired-end sequencing. Reads generated across the three lanes were merged and demultiplexed using Illumina software bcl2fastq v1.8.4, allowing no mismatches and excluding the last position (eighth index base).

Paired-end reads were mapped to the S288c *S*. *cerevisiae* genome R64-2-1 [42,109] with ERCC spike-in sequences added using BWA mem Version: 0.7.12-r1039 and default parameters [109]. Reads were processed with Picard tools Version: 1.98(1547) cleansam and AddOrReplaceReadGroups as required by downstream applications. Resulting bam files were sorted and indexed using Samtools Version 1.2. Paired-end fragments were deduplicated using the RemoveDuplicate function in Picardtools, and read counts mapped to genes were extracted using FeatureCounts Version 1.5.0. Sequenced wells were removed from the analysis if they had <1,000 total mapped reads or if the proportion of ERCC spike-ins to total-mapped reads was > 0.2 [110]. Data were normalized by SCNorm [111] in R version 3.3.1; ERCC spike-in samples were not used in the normalization. Normalized read counts for each gene were logged and then centered by subtracting the mean log_2_(read counts) for that gene across all cells in the analysis. All data are available in the NIH GEO database under access number GSE102475.

### Statistical analysis of differential expression

Genes defined in iESR, RP, and RiBi clusters [24] are annotated in S8 Table. Individual cells with altered expression of defined gene groups (e.g., cells with low RP expression as in Fig 1C or high Rpn4/Hsf1 targets as in Fig 6) were determined using a two-tailed Welch *t* test, comparing the set of mean-centered log_2_(read count) values in that cell to the combined set of mean-centered log_2_(read count) values for all other unstressed (or stressed) cells, taking FDR <5% [112] as significant. Significance for Fig 2 was determined in two ways: by Welch *t* test on logged CV for RP, iESR, and RiBi gene groups compared to all other genes and by random sampling without replacement to compare median CV values for each group; for this analysis, only transcripts measured in at least four cells were assessed (since CV may be artificially high for poorly measured transcripts). CV of RP-cluster mRNAs was significantly lower than other transcripts, by *t* test (*p* = 2e-59 and *p* = 3e-22 for unstressed and stressed cells, respectively) and by random sampling (*p* < 1e-6 for both unstressed and stressed cells). CV for iESR transcripts was significantly lower than other transcripts after stress (*p* = 8e-7 by *t* test and *p* < 1e-6 by sampling), although the effect was subtle (median CV = 0.90 versus 1.06 for other transcripts). iESR transcripts from unstressed cells and RiBi transcripts from stressed cells showed very slightly (approximately 3%) higher median CV that was significant by *t* test (*p* = 6e-5 and *p* = 7e-5, respectively) but not sampling (*p* = 0.15 and *p* = 0.0516, respectively).

To score differential expression of ESR groups across cell-cycle phases, a two-tailed Welch *t* test was applied to the mean-centered log_2_(read count) values, comparing the pooled set of values from all cells in a given cell-cycle phase to the pooled values from unstressed or stressed cells from all other phases; stressed and unstressed cells were analyzed separately unless otherwise noted. All cell classifications from this work are summarized in S9 Table.

The Oscope [64] R package version 1.4.0 was used to identify oscillatory genes in the set of unstressed cells. Oscope first filtered transcripts using the function CalcMV and analyzed only those having a minimum mean larger than 15 (MeanCutLow = 15 and otherwise default parameter settings). Oscope then fit sinusoidal functions to all remaining mRNA pairs, and those identified as oscillating were clustered according to their oscillation frequencies. Oscope was also run with relaxed mean and variance thresholds to consider all genes with a mean larger than 10 and the maximum number of clusters set to five in the K-medioids clustering step (S4 Table). Oscope computationally reordered the single-cells for the two detected gene clusters. The cyclic orderings were used to identify additional genes following the same orders by fitting a third-degree polynomial to all genes using the WaveCrestIden function from the WaveCrest [66] R package version 0.0.1. Genes were ranked by their fit using the mean squared error (MSE), and only the top 100 genes were considered further. The WaveCrestIden function was run twice, either including zeros in the polynomial fit or treating them as missing. The WaveCrest algorithm was also used as above to obtain a cyclic order on the set of unstressed single cells based on five RP genes (S5 Table).

### Variance and detection-rate analyses

Length-normalized read counts were taken as SCNorm-normalized read counts per transcript divided by transcript length, and the mean (or median) for each transcript across all unstressed or stressed cells was calculated; mean and median values were essentially the same (R^2^ = 0.99). Unless otherwise noted, transcripts with no read count in that sample were not included in the calculation (instead of counting the value as 0)—the only exception was in calculating correlations between average scRNA-seq data compared to bulk data (e.g., S4 Fig), for which the correlation was significantly higher by scoring no-read transcripts as zero values and including them in the calculation. Detection rate was defined as the fraction of unstressed or stressed cells in which a gene was detected by at least one collapsed read count. We devised a statistic to test if RP, RiBi, or iESR gene groups were significantly different from other genes. For each set of genes, a cubic smoothing spline was fit to describe the relationship between detection rate and median expression, and the point along the curve at which 80% of points were fit was identified. This process was repeated for 10,000 random gene sets equal to the size of the query gene group. The *p*-value was calculated as the fraction of random gene sets having a statistic more extreme than the observed value. The calculated statistic for unstressed and stressed cells, respectively, was: RP: 0.045, 0.089; RiBi: 0.148, 0.156; iESR: 0.493, 0.208. RP genes were also compared against 10,000 trials randomly selecting mRNAs shorter than the median-RP gene length. Tests were repeated on genes without a close homolog in the S288c genome (i.e., genes with BLAST hits of E > 1e−5). All significant tests shown in Fig 3 remained significant (*p* < 0.05) except for RP transcripts after stress (*p* = 0.36).

### Cell classifications and gene clustering

Data were clustered with Pagoda [54] using default parameters, and clusters enriched for known cell-cycle regulators [65] or glycolysis transcripts were manually identified. We were unable to identify known cell-cycle markers that peaked in M phase, either in the Pagoda-clustered data or using known M-phase markers [65]. To classify cells according to cell-cycle phase, cells were organized by hierarchical clustering [113] based on the centroid (median) of mean-centered log_2_(transcript abundance) for transcripts in each cell-cycle phase (S3 Table) and manually sorted and classified based on overlapping peaks of each vector (Fig 5A). Cells that showed no expression peak in any of the vectors were scored as “unclassified”.

### Identification of TF target expression differences

Compiled lists of TF targets were taken from [93]. We added to this an additional list of Msn2 targets, taken as genes with stress-dependent Msn2 binding within the 800 bp upstream region and whose normal induction during peroxide stress required *MSN2/MSN4* [114] (S7 Table) and genes whose repression requires the *DOT6/TOD6* repressors (defined here as genes with a 1.5X repression defect in two biological replicates of wild-type BY4741 and *dot6Δtod6Δ* cells responding to 0.7M NaCl for 30 min [53] (S7 Table). In total, we scored 623 overlapping sets of TF targets defined in various datasets [114–117] or summarized from published ChIP studies [118]. We identified TF targets with concerted expression changes in two ways. First, we identified the distribution tails in each cell, identifying all mRNAs in that cell whose mean-centered log_2_(read count) was ≥1.0 or ≥2.0 (i.e., two times or four times higher than the population mean). We then used the hypergeometric test to identify sets of TF targets enriched on either list, taking the lower of the two *p*-values for that TF–cell comparison. Comparable tests were done for the sets of genes whose relative abundance was ≤−1.0 or ≤−2.0 in each cell. TFs with −log_10_(*p*-value) > 4 were taken as significant (equivalent to a cell-based Bonferroni correction, *p* = 0.05 / 623 tests = approximately 1e-4). We focused on TFs whose targets were enriched at the distribution tails in at least four cells, which is unlikely to occur by chance. Two sets of TF targets were removed because their targets heavily overlapped with ESR targets and their enrichment was not significant when those overlapping mRNAs were removed (S6 Table). In a second approach, we used Welch *t* tests to compare the mean-centered log_2_(read count) values of each set of TF targets compared to all other measured mRNAs in that cell, again taking −log_10_(*p*-value) > 4 as significant and focusing on TFs identified in at least three cells. Finally, for Fig 6E and 6F, colored boxplots indicate TF targets whose relative abundances in the denoted cell were significantly different from all other stressed cells (FDR < 0.053).

### sm-FISH

BY4741 was grown as described above and collected for fixation and processing as previously described [119]. FISH probes were designed using the Biosearch Technologies Stellaris Designer with either Quasar 670, CAL Fluor Red 610, or Quasar 570 dye, using 33 probes for *PPT1* (Quasar 670) and *RLP7* (Quasar 570) and 48 probes for *SES1* (Quasar 570). *PPT1* and *RLP7* were measured in the same cells, and *SES1* was measured in a separate experiment as a control. Images were acquired as z-stacks every 0.2 mm with an epifluorescent Nikon Eclipse-TI inverted microscope using a 100x Nikon Plan Apo oil immersion objective and Clara CCD camera (Andor DR328G, South Windsor, Connecticut, United States of America). Quasar 670 emission was visualized at 700 nm upon excitation at 620 nm (Chroma 49006_Nikon ET-Cy5 filter cube, Chroma Technologies, Bellows Falls, Vermont, USA). Quasar 570 emission was visualized at 605 nm upon excitation at 545 nm (Chroma 49004_Nikon ET-Cy3 filter cube). *PPT1* and *RLP7* transcripts were counted manually, while *SES1* mRNA was counted by semiautomated transcript detection and counting in MATLAB using scripts adapted from [120].

### Fixed-cell microscopy

Cells were grown as described above and fixed with 3.7% formaldehyde for 15 min either before or at indicated times after NaCl addition. Cells were washed two times with 0.1 M potassium phosphate buffer pH 7.5, stained 5 min with 1 μg/mL DAPI (Thermo Scientific Pierce, 62247), and additionally washed two times with 0.1 M potassium phosphate buffer pH 7.5. Cells were imaged on an epifluorescent Nikon Eclipse-TI inverted microscope using a 100x Nikon Plan Apo oil immersion objective. GFP emission was visualized at 535 nm upon excitation at 470 nm (Chroma 49002_Nikon ETGFP filter cube, Chroma Technologies, Bellows Falls, VT, USA). mCherry emission was visualized at 620 nm upon excitation at 545 nm (Chroma 96364_Nikon Et-DSRed filter cube). DAPI emission was visualized at 460 nm upon excitation at 350 nm (Chroma 49000_Nikon ETDAPI filter cube). Nuclear to cytoplasmic intensity values were calculated with customized CellProfiler scripts [121]. The fraction of cells with nuclear factor before stress was calculated by identifying nuclear masks in Cell Profiler (i.e., identifiable nuclear objects) in that channel that overlapped with DAPI masks and manually correcting miscalls.

### Live-cell microscopy

Yeast strains AGY1328 (Dot6-GFP, Msn2-mCherry) and AGY1331 (Sfp1-GFP, Msn2-mCherry) were grown at 30 °C with shaking to OD_600_ 0.4–0.5 in low-fluorescence yeast medium (LFM) [119]; the fraction of cells with nuclear Msn2/Dot6/Sfp1 was very similar in fixed, unstressed cells growing in LFM versus YPD, not shown. Cells were imaged in a Focht Chamber System 2 (FCS2) (Bioptechs, Inc; Butler, Pennsylvania, USA) with temperature maintained at 30 °C. Cells were loaded into the chamber and adhered to a 40-mm round glass coverslip. Briefly, the coverslip was prepared by incubating with concanavalin A solution (two mg/ml in water) for two min at room temperature. The concanavalin A was aspirated, and 350 μl of cell culture added. Cells were allowed two min at room temperature to adhere to the concanavalin A before the gasket was washed once with 350 μl of fresh LFM media. A round 0.2-μM gasket contained the concanavalin A plus cell solution. The entire assembly, including coverslip, cells, and gasket, was assembled with the microaqueduct slide, upper gasket, and locking base—as per manufacturer instructions—to create an incubated perfusion chamber. This assembly was transferred to an epifluorescent Nikon Eclipse-TI inverted microscope for time-lapse imaging. Temperature was maintained at 30 °C using the Bioptechs temperature controller. Steady perfusion with LFM or LFM + 0.7 M NaCl (initiated at 81 min) was maintained utilizing microperfusion pumps feeding media through the FCS2 perfusion ports. Once loaded onto the microscope, cells and temperature were monitored for at least one hr to ensure stable temperature and robust growth.

During each timecourse experiment, a TI-S-ER motorized stage with encoders (Nikon MEC56100) and PerfectFocus system (Nikon Instruments, Melville, New York, USA) were used to monitor specific stage positions and maintain focus throughout the timecourse. A Clara CCD Camera (Andor DR328G, South Windsor, Connecticut, USA) was used for imaging. GFP emission was visualized at 535 nm (50-nm bandwidth) upon excitation at 470 nm (40-nm bandwidth; Chroma 49002_Nikon ETGFP filter cube, Chroma Technologies, Bellows Falls, Vermont, USA). mCherry emission was visualized at 620 nm (60-nm bandwidth) upon excitation at 545 nm (30-nm bandwidth; Chroma 6364_Nikon Et-DSRed filter cube). Single-cell traces of nuclear localization were extracted from fluorescence images using custom Fiji [122] and Matlab scripts. Fiji was used to threshold and identify individual cells. Individual cell traces were constructed using a modified Matlab particle tracking algorithm [123] and then manually validated and corrected. Localization of individual transcription factors was quantified using a previously published localization score based on the difference between the mean intensity of the top 5% of pixels in the cell and the mean intensity of the other 95% of pixels in the cell [124]. Peaks of nuclear TF localization were identified using findpeaks2 (Matlab File Exchange).

## Supporting information

S1 Table Statistics on sequencing features. (XLSX)

S2 Table Transcripts outside RP-splines and iESR-fit splines. iESR, induced-Environmental Stress Response; RP, ribosomal protein.
(XLSX)

S3 Table Cell-cycle expression vectors and gene lists used for classification. (XLSX)

S4 Table Oscope-identified clusters of cycling genes. (XLSX)

S5 Table Wavecrest-identified transcripts associated with RP mRNAs. RP, ribosomal protein.
(XLSX)

S6 Table Specific TF-target gene sets identified in Fig 6 analysis. TF, transcription factor.
(XLSX)

S7 Table Lists of all TF targets considered for Fig 5 analysis. TF, transcription factor.
(XLSX)

S8 Table Lists of genes included in iESR, RiBi, and RP clusters used in the analysis. iESR, induced-Environmental Stress Response; RiBi, ribosome biogenesis; RP, ribosomal protein.
(XLSX)

S9 Table Summary of all cell classifications. (XLSX)

S10 Table FISH counts plotted in Fig 4B. Counts from unstressed and stressed cells are recorded for different transcripts on different tabs of the file. *PPT1* and *RLP7* were measured in the same cells; *SES1* was measured alone in a different set of cells. FISH, fluorescence in situ hybridization.
(XLSX)

S11 Table Nuclear versus cytosolic TF localization plotted in Fig 7A. Nuclear/cytosolic ratios from Cell Profiler are shown for each of two factors as measured in a single strain. Each strain data is shown on a separate tab. TF, transcription factor.
(XLSX)

S12 Table Ratios from Fig 7C, as described in the text. (XLSX)

S1 Fig Mean iESR and RiBi transcript levels in cells with low RP mRNA. For each cell plotted, the median of the mean-centered log_2_(read count) values for the group of RP transcripts was plotted against the median of the mean-centered log_2_ (read count) values for (A) iESR transcripts and (B) RiBi transcripts in that cell. Red points represent the cells in which iESR transcripts were concertedly high (Fig 1C, asterisks). The RiBi-transcript detection rate (percentage of transcripts measured) for each cell is represented above the corresponding points in B, color-coded according to the key. R^2^ is shown for all points and excluding red points in which iESR transcripts were relatively high. iESR, induced-Environmental Stress Response; RiBi, ribosome biogenesis; RP, ribosomal protein.
(TIF)

S2 Fig iESR, RP, and RiBi abundance by cell-cycle phase. Boxplots (without whiskers) of (A) RP, (B) iESR, (C) RiBi mRNAs for cells classified in different cell-cycle phases. Each boxplot represents the distribution of relative mRNA abundances, as defined in Methods, within each cell. Significance was assessed by Welch *t* test on the pooled RP, iESR, or RiBi genes from cells within a given phase compared to the pooled set of those transcripts from all other cells; unstressed and stressed cells were analyzed separately. Dark blue boxplots in (A) represent significant groups, with FDR listed below. RP expression was slightly, but highly statistically significantly, higher among cells in G1 phase, particularly for subsets of cells. A subset of unstressed cells in S-phase showed particularly low mean-centered log_2_ abundance of RP transcripts. In stressed cells, those in S-phase had particularly tight distribution of RP abundance, showing in effect weaker RP repression during stress than cells in other phases. There were no significant differences across cell-cycle phases for expression of iESR or RiBi transcripts. iESR, induced-Environmental Stress Response; RiBi, ribosome biogenesis; RP, ribosomal protein.
(TIF)

S3 Fig No clear evidence for the YMC. (A) Mean-centered log_2_(read count) values of several groups of transcripts, in unstressed cells with low RPs, other unstressed cells, and stressed cells, as described in Fig 1C. Cells are ordered as in Fig 1, except that unstressed, low-RP cells are displayed first. Gene groups include: 149 mRNAs annotated as part of the ESR RP cluster, 28 genes identified by Pagoda clustering that are heavily enriched for glycolysis transcripts, 23 and 40 YMC-related mRNAs identified by Tu *et al*. enriched for mitochondrial or peroxisomal (POX) functions; also shown are 82 transcripts encoding mitochondrial ribosomal proteins. (B) Boxplots (without whiskers) of the distribution of mean-centered log_2_(read counts) for the group of Pagoda-identified glycolysis transcripts, in individual cells ordered as in Fig 1C. Cells with statistically different expression of glycolysis mRNAs (FDR < 0.05) are highlighted in dark blue (unstressed and stressed cells were analyzed separately). Cells with lower RP expression from Fig 1C are indicated with arrows and asterisks, as described in Fig 1C. Cells with low-RP expression had no statistically significant difference in glycolysis-mRNA expression. ESR, Environmental Stress Response; POX, peroxisomal; RP, ribosomal protein; YMC, yeast metabolic cycle.
(TIF)

S4 Fig Correlation between scRNA-seq and bulk RNA-seq for specific gene sets. Correlation between bulk RNA-seq log_2_(fold-change) after 0.7 M NaCl and average of single cell measurements. Bulk data represent the average of three biological replicates. R^2^ is shown for each plot. Correlations were substantially higher when transcripts with no reads in a given cell were scored as zero reads (rather than treated as missing values), which supports the notion that missing measurements are influenced biological variation in transcript presence per cell, rather than purely noise specific to scRNA-seq data. RNA-seq, RNA sequencing; scRNA-seq, single-cell RNA sequencing.
(TIF)

S5 Fig Genes with more upstream Msn2 sites do not show significant differences in pre-stress activation. The number of Msn2 binding sites (CCCCT or CCCCC) within 800bp upstream of the gene starts was identified in Msn2 targets defined by Huebert et al (2012). The distribution of mean-centered log_2_(read counts) in unstressed cells for genes with at least five upstream elements (left) was not statistically significantly different from genes with one or two upstream elements (right) (Welch *t* test comparing the two groups in each cell). Cells are ordered as in Fig 1C. None of the cells showed concerted differences in Msn2 target expression before stress (FDR < 0.05, Welch *t* test, see Methods). There was no relationship between the median relative log_2_ read counts and cells with low RPs.
(TIF)

S1 Data Representative timecourse of Msn2-mCherry and Sfp1-GFP before and after addition of 0.7 M NaCl. The pre-stress phase is indicated by a white scale bar, which turns red upon NaCl addition. We were unable to call troughs of nuclear Sfp1-GFP before stress. Apparent Sfp1-GFP nuclear depletion, without nuclear Msn2-mCherry during the unstressed phase, is seen in the cell at the far upper left corner; the nucleus is clearly in focus judged by the nuclear Msn2-mCherry concentration upon NaCl treatment. GFP, green fluorescent protein.
(AVI)

S2 Data Representative timecourse of Msn2–mCherry and Dot6–GFP before and after addition of 0.7M NaCl. The pre-stress phase is indicated by a white scale bar, which turns red upon NaCl addition. GFP, green fluorescent protein
(AVI)

## Abbreviations

BLAST: Basic Local Alignment Search Tool
CV: coefficient of variance
ESR: Environmental Stress Response
FCS2: Focht Chamber System 2
FDR: false discovery rate
GFP: green fluorescent protein
iESR: induced-Environmental Stress Response
LFM: low-fluorescence yeast medium
M-phase: mitosis-phase
MSE: mean squared error
PKA: Protein Kinase A
rESR: repressed-Environmental Stress Response
RiBi: ribosome biogenesis
RNA-seq: RNA sequencing
RP: ribosomal protein
scRNA-seq: single-cell RNA sequencing
sm-FISH: single-molecule fluorescence in situ hybridization
TF: transcription factor
YMC: yeast metabolic cycle
