# Supplementary figures and images for: Single-cell RNA sequencing reveals intrinsic and extrinsic regulatory heterogeneity in yeast responding to stress
Source: PLoS Biol. 2017 Dec 14;15(12):e2004050. doi: 10.1371/journal.pbio.2004050 (PMC5746276; doi:10.1371/journal.pbio.2004050)

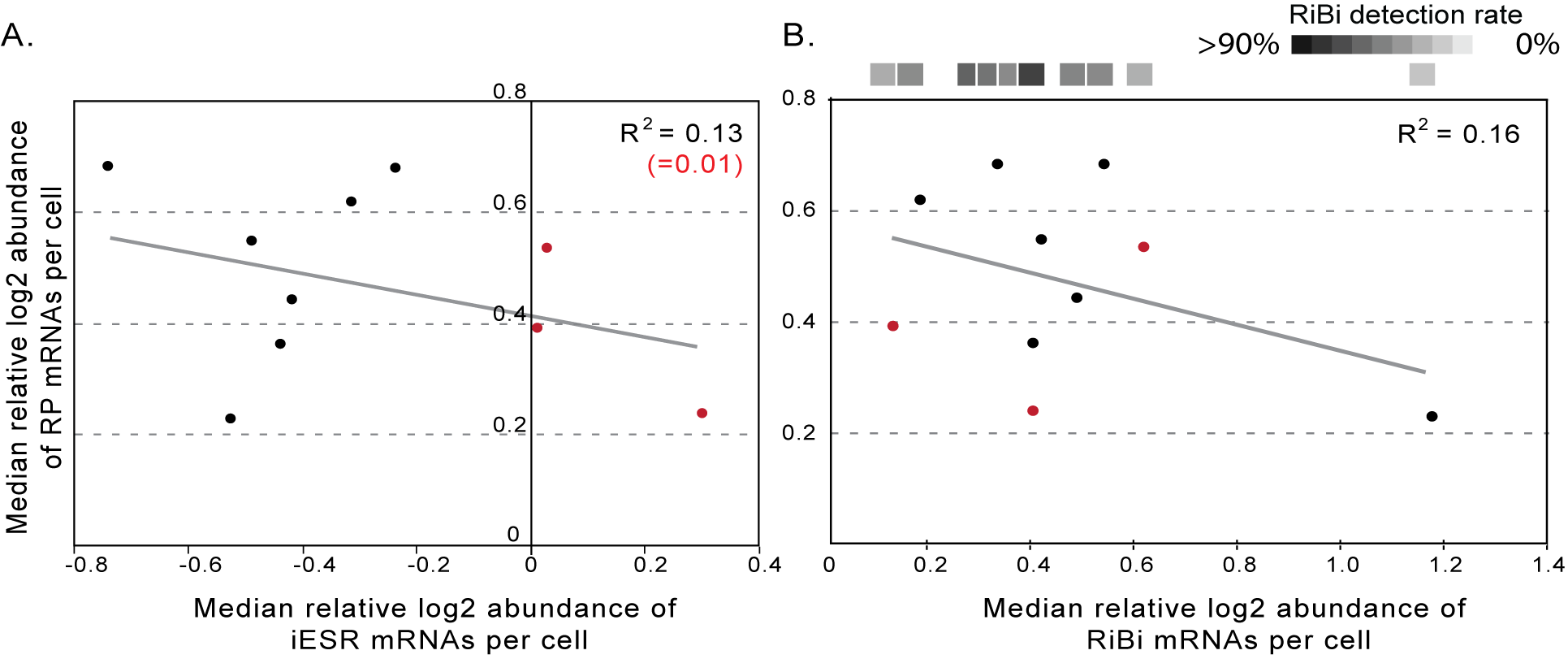

Supplement: S1 Fig — For each cell plotted, the median of the mean-centered log2(read count) values for the group of RP transcripts was plotted against the median of the mean-centered log2 (read count) values for (A) iESR transcripts and (B) RiBi transcripts in that cell. Red points represent the cells in which iESR transcripts were concertedly high (Fig 1C, asterisks). The RiBi-transcript detection rate (percentage of transcripts measured) for each cell is represented above the corresponding points in B, color-coded according to the key. R2 is shown for all points and excluding red points in which iESR transcripts were relatively high. iESR, induced-Environmental Stress Response; RiBi, ribosome biogenesis; RP, ribosomal protein. (TIF) [file pbio.2004050.s013.tif]

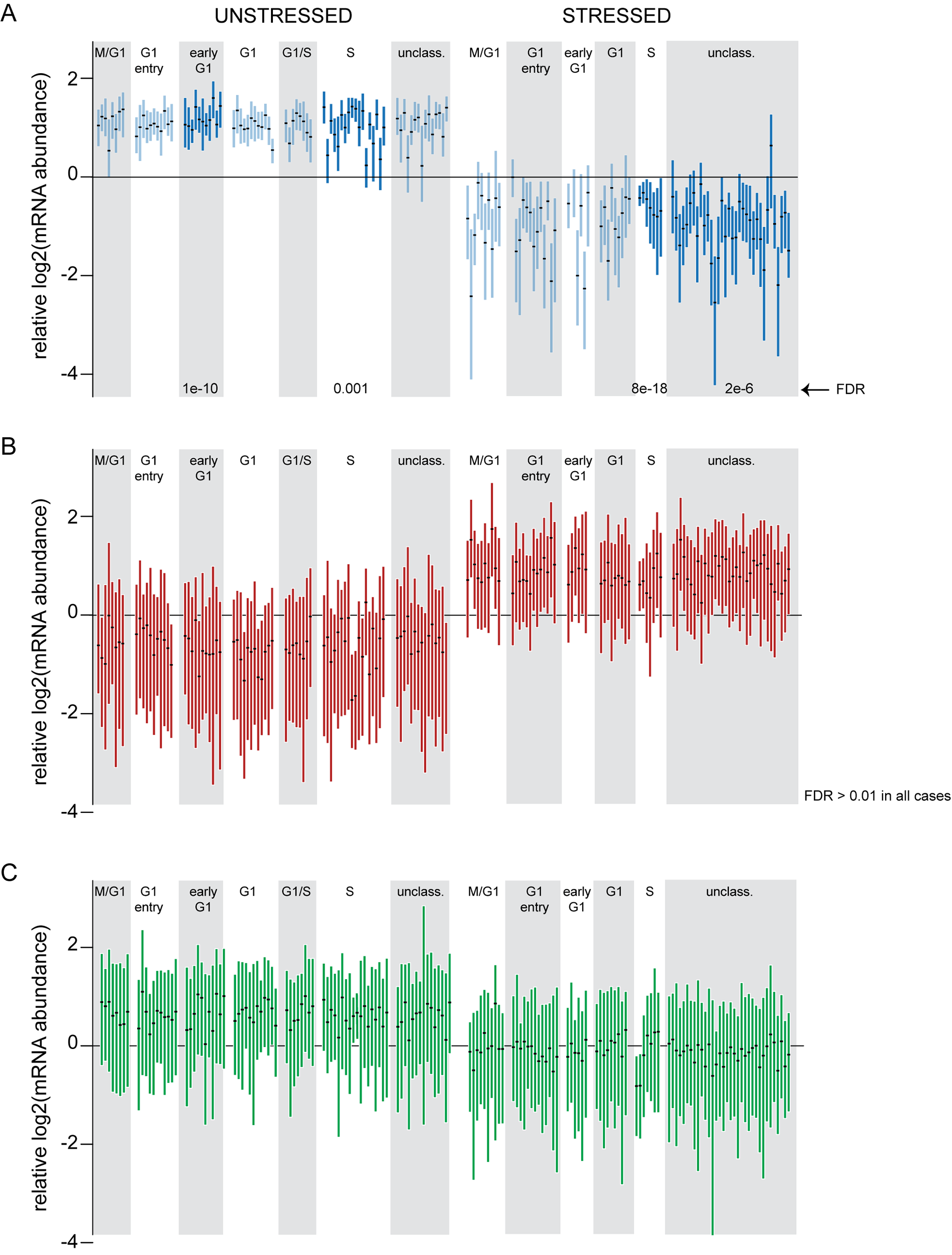

Supplement: S2 Fig — Boxplots (without whiskers) of (A) RP, (B) iESR, (C) RiBi mRNAs for cells classified in different cell-cycle phases. Each boxplot represents the distribution of relative mRNA abundances, as defined in Methods, within each cell. Significance was assessed by Welch t test on the pooled RP, iESR, or RiBi genes from cells within a given phase compared to the pooled set of those transcripts from all other cells; unstressed and stressed cells were analyzed separately. Dark blue boxplots in (A) represent significant groups, with FDR listed below. RP expression was slightly, but highly statistically significantly, higher among cells in G1 phase, particularly for subsets of cells. A subset of unstressed cells in S-phase showed particularly low mean-centered log2 abundance of RP transcripts. In stressed cells, those in S-phase had particularly tight distribution of RP abundance, showing in effect weaker RP repression during stress than cells in other phases. There were no significant differences across cell-cycle phases for expression of iESR or RiBi transcripts. iESR, induced-Environmental Stress Response; RiBi, ribosome biogenesis; RP, ribosomal protein. (TIF) [file pbio.2004050.s014.tif]

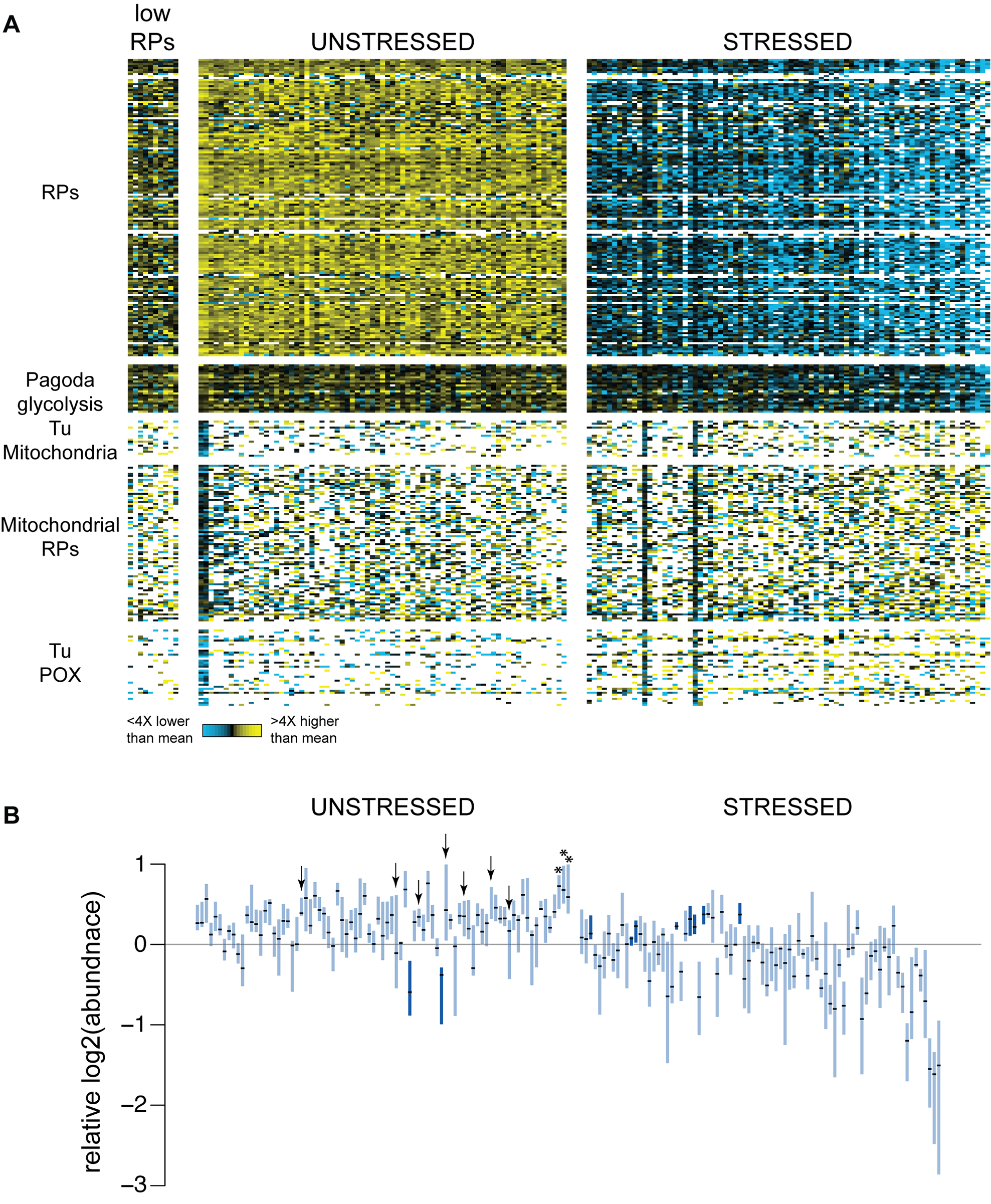

Supplement: S3 Fig — (A) Mean-centered log2(read count) values of several groups of transcripts, in unstressed cells with low RPs, other unstressed cells, and stressed cells, as described in Fig 1C. Cells are ordered as in Fig 1, except that unstressed, low-RP cells are displayed first. Gene groups include: 149 mRNAs annotated as part of the ESR RP cluster, 28 genes identified by Pagoda clustering that are heavily enriched for glycolysis transcripts, 23 and 40 YMC-related mRNAs identified by Tu et al. enriched for mitochondrial or peroxisomal (POX) functions; also shown are 82 transcripts encoding mitochondrial ribosomal proteins. (B) Boxplots (without whiskers) of the distribution of mean-centered log2(read counts) for the group of Pagoda-identified glycolysis transcripts, in individual cells ordered as in Fig 1C. Cells with statistically different expression of glycolysis mRNAs (FDR < 0.05) are highlighted in dark blue (unstressed and stressed cells were analyzed separately). Cells with lower RP expression from Fig 1C are indicated with arrows and asterisks, as described in Fig 1C. Cells with low-RP expression had no statistically significant difference in glycolysis-mRNA expression. ESR, Environmental Stress Response; POX, peroxisomal; RP, ribosomal protein; YMC, yeast metabolic cycle. (TIF) [file pbio.2004050.s015.tif]

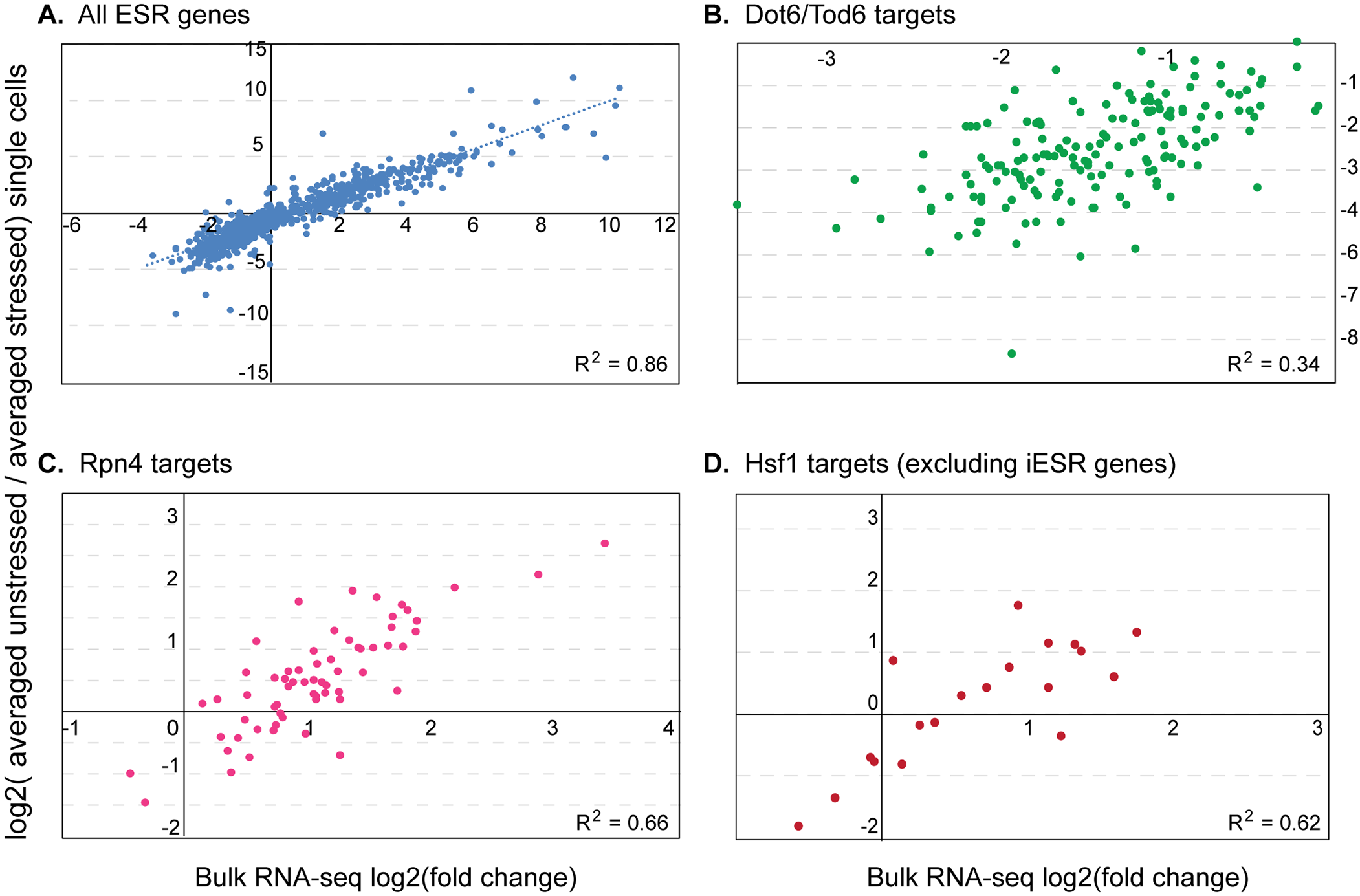

Supplement: S4 Fig — Correlation between bulk RNA-seq log2(fold-change) after 0.7 M NaCl and average of single cell measurements. Bulk data represent the average of three biological replicates. R2 is shown for each plot. Correlations were substantially higher when transcripts with no reads in a given cell were scored as zero reads (rather than treated as missing values), which supports the notion that missing measurements are influenced biological variation in transcript presence per cell, rather than purely noise specific to scRNA-seq data. RNA-seq, RNA sequencing; scRNA-seq, single-cell RNA sequencing. (TIF) [file pbio.2004050.s016.tif]

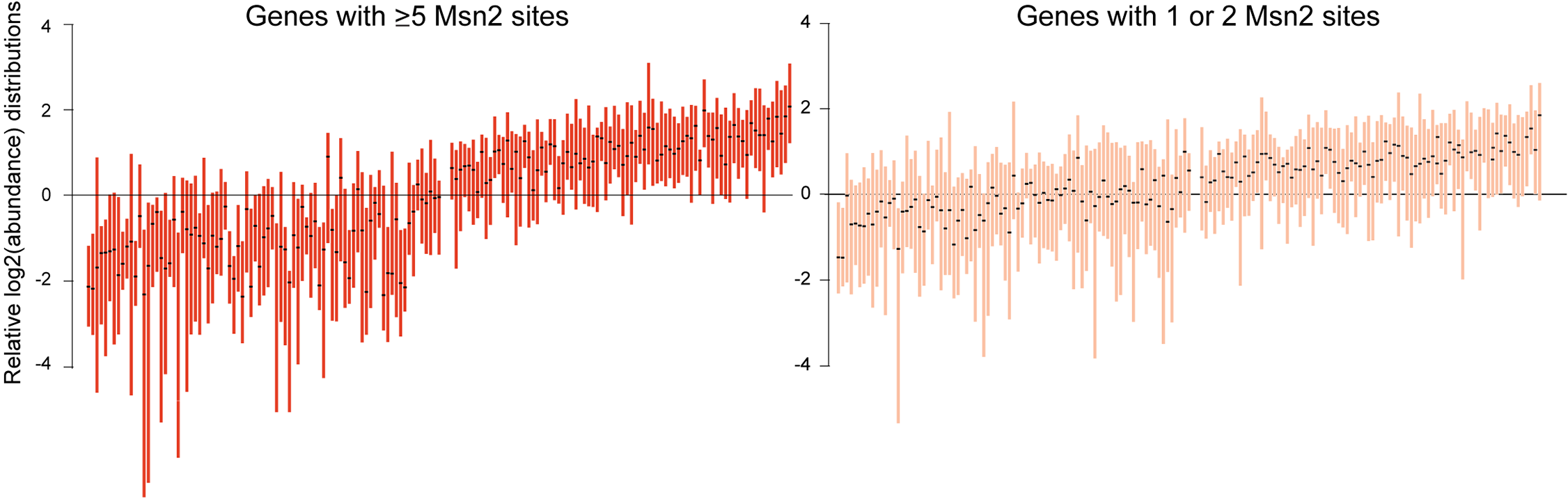

Supplement: S5 Fig — The number of Msn2 binding sites (CCCCT or CCCCC) within 800bp upstream of the gene starts was identified in Msn2 targets defined by Huebert et al (2012). The distribution of mean-centered log2(read counts) in unstressed cells for genes with at least five upstream elements (left) was not statistically significantly different from genes with one or two upstream elements (right) (Welch t test comparing the two groups in each cell). Cells are ordered as in Fig 1C. None of the cells showed concerted differences in Msn2 target expression before stress (FDR < 0.05, Welch t test, see Methods). There was no relationship between the median relative log2 read counts and cells with low RPs. (TIF) [file pbio.2004050.s017.tif]
